# Allogeneic CAR-engineered cellular therapy for relapsed and refractory large B cell lymphoma: a systematic review and meta-analysis

**DOI:** 10.3389/fimmu.2025.1585556

**Published:** 2025-07-08

**Authors:** Alexander Biederstädt, Florian Bassermann, Judith S. Hecker

**Affiliations:** ^1^ Department of Medicine III, Technical University of Munich (TUM), School of Medicine and Health, Munich, Germany; ^2^ TranslaTUM, Center for Translational Cancer Research, Technical University of Munich (TUM), Munich, Germany; ^3^ Deutsches Konsortium für Translationale Krebsforschung (DKTK), Heidelberg, Germany; ^4^ Bavarian Cancer Research Center (BZKF), Munich, Germany

**Keywords:** allogeneic CAR-T cells, allogeneic CAR-NK cells, adoptive cell therapy (ACT), cellular engineering, CRISPR gene editing, precision gene editing, large B cell lymphoma, clinical trial

## Abstract

**Introduction:**

Relapsed/refractory (r/r) large B-cell lymphoma (LBCL) remains a difficult-to-treat disease with limited treatment options and high unmet clinical need, necessitating the development of new therapies with greater potency and broader applicability. While autologous chimeric antigen receptor (CAR)-T cell therapies have transformed the treatment landscape, 60–65% of patients receiving these therapies eventually relapse, underscoring the need for improved approaches. Allogeneic CAR-T and CAR-NK cell therapies have recently emerged as promising alternatives, offering the potential to shorten manufacturing times, reduce costs, and expand access to a broader patient population. This systematic review and meta-analysis compiles the currently available clinical trial data on the efficacy and safety of these novel therapies in adult patients with r/r LBCL.

**Methods:**

A systematic search of MEDLINE, EMBASE, Web of Science, and the Cochrane Central Register of Controlled Trials was conducted for studies published up to January 12, 2025, involving allogeneic CAR-T and CAR-NK cell therapies in R/R LBCL. The primary outcomes assessed were the best overall response rate (bORR) and best complete response rate (bCRR) at any time point. Secondary outcomes included rates of grade 1-2 and grade 3+ cytokine release syndrome (CRS), grade 1-2 and grade 3+ immune effector cell-associated neurotoxicity syndrome (ICANS), grade 1-2 and grade 3+ infections and incidence of graft-versus-host disease (GvHD).

**Results:**

Nineteen studies met the inclusion and exclusion criteria, encompassing 334 patients (155 CAR-NK; 179 CAR-T) evaluable for safety and 235 patients evaluable for response (77 CAR-NK; 158 CAR-T). The pooled estimates for the best overall response rate (bORR) and the best complete response rate (bCRR) were 52.5% [95% CI, 41.0-63.9] and 32.8% [95% CI, 24.2-42.0], respectively. Safety analysis revealed very low incidences of grade 3+ CRS (0.04% [95% CI 0.00-0.49]) or grade 3+ ICANS (0.64% [95% CI 0.01-2.23]) and only one occurrence of a GvH-like reaction across 334 infused patients enrolled in the included studies, highlighting the remarkable safety profile of CAR-engineered “off-the-shelf” allogeneic approaches. The estimated overall incidence of low-grade CRS was 30% [95% CI, 14-48], while the estimated overall incidence of low-grade ICANS was 1% [95% CI, 0%-4%], markedly lower than current-generation autologous CAR-T cell products. The incidence of low-grade and severe infections was 25% [95% CI 14-36%) (n=252) and 7% [95% CI 2-14%] (n=291), respectively.

**Discussion:**

Together, allogeneic CAR-T and CAR-NK cell therapies demonstrate encouraging efficacy in heavily pretreated patients with r/r LBCL. Coupled with their favorable safety profiles and the potential for off-the-shelf availability, allogeneic cell therapies hold great promise to broaden the reach of live cell-based treatments, delivering impactful results to a wider patient population in the coming years.

## Graphical Abstract

Allogeneic CAR-engineered immune effector cells (CAR-IEC) for relapsed and refractory large B cell lymphoma (r/r LBCL).

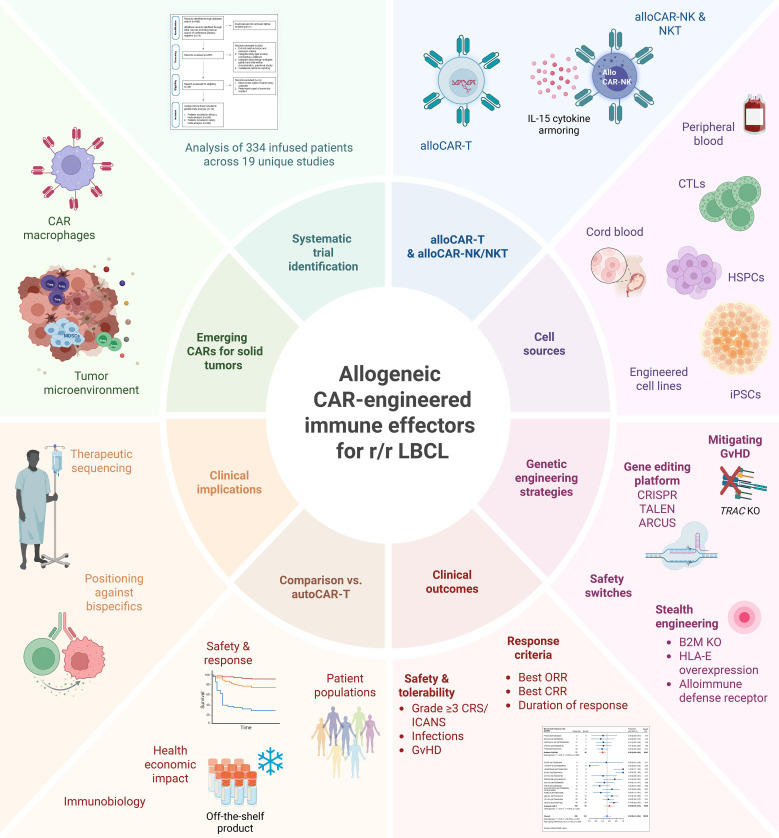

## Introduction

Chimeric antigen receptor (CAR)-T cell therapy has revolutionized the treatment landscape for relapsed and refractory (r/r) hematologic malignancies, including CD19-positive lymphoid malignancies and multiple myeloma. Over the past decade, seven FDA-approved autologous CAR-T cell products have reached clinical use, each targeting specific indications for patients with r/r disease (1–15). These advances represent a major milestone in oncology, showcasing the potential of cellular immunotherapies.

Long-term and real-world evidence has begun to validate the transformative potential of CAR-T therapy, with durable responses observed in a significant fraction of patients (6, 8, 9, 16). Notably, recent studies have detected CAR-positive T cells persisting up to 10 years post-infusion, underscoring the durability and adaptability of these engineered cellular therapies (17). Despite these remarkable successes, significant challenges remain. Many patients treated with current-generation CAR-T cell products fail to achieve durable responses, and a substantial proportion ultimately relapse (1, 10, 15, 16, 18, 19).

Moreover, the reliance on autologous CAR-T cells introduces logistical and biological challenges. The need for patient-specific lymphocyte apheresis and the complexities of individualized manufacturing extend time to treatment and increase costs. Efforts to decentralize manufacturing of autologous CAR-T cells are being explored to mitigate some of the logistical and cost hurdles (20). However, decentralized manufacturing cannot overcome the fundamental challenge posed by patients with low lymphocyte counts or dysfunctional lymphocytes, which may result from prior chemoimmunotherapy, tumor-induced immunosuppression, or inherent T cell defects, such as those seen in HIV-associated DLBCL. For such patients, allogeneic CAR-T and CAR-NK therapies, which utilize healthy donor-derived or induced pluripotent stem cell (iPSC)-derived cells, may provide a distinct advantage by circumventing the need for patient-derived starting material (21–36).

Recently, the development of bispecific antibodies targeting both CD3 on T cells and tumor antigens such as CD19 and CD20 has provided a potential alternative to autologous CAR-T therapy. These therapies are logistically simpler to deliver, as they are immediately available and typically associated with lower-grade toxicities, particularly in terms of CRS and ICANS, compared to CAR-T cell therapy (37, 38). However, bispecific antibodies are similarly reliant on the fitness of the recipient’s immune system to exert their effects and, unlike CAR-T cells, require extended or indefinite dosing regimens. Furthermore, although direct comparisons between bispecific antibodies and CAR-T cells are limited, observed response rates with bispecific antibodies are generally lower than those achieved with CAR-T cells in similar treatment settings (37, 38). Thus, while bispecific antibodies represent an important addition to the therapeutic armamentarium, they underscore the pressing need for advancements that enhance the efficacy and accessibility of the CAR-T platform.

To address these limitations, the development of novel CAR-equipped allogeneic cell therapies has gained significant momentum. Allogeneic approaches leverage lymphocytes from healthy donors or clonal iPSC master cell lines, enabling the creation of “off-the-shelf” products that bypass the need for patient-specific manufacturing. These cryopreserved therapies are readily available for point-of-care administration, streamlining the treatment process. Furthermore, the use of lymphocytes from healthy donors circumvents issues related to compromised cellular fitness and expands the therapeutic potential. Different lymphocyte sources have been explored for allogeneic CAR engineering, each offering distinct advantages and limitations.

Among the most studied are αβ T cells, which provide robust cytotoxic activity and the potential for immune memory formation, making them highly effective in targeting malignancies. However, the presence of endogenous T-cell receptors (TCRs) in αβ T cells poses a risk of graft-versus-host disease (GvHD), necessitating complex genetic modifications to knock out the TCR (26, 27, 32, 33, 35, 36). Another emerging option are γδ T cells, which blend adaptive and innate immune properties, exhibiting potent anti-tumor activity while demonstrating a reduced risk of GvHD. However, their lower prevalence in peripheral blood and complex biology pose challenges for large-scale clinical application (34).

Natural killer (NK) cells, by contrast, do not express TCRs eliminating the risk of TCR-mediated GvHD, thereby simplifying the engineering process. NK cells also possess innate anti-tumor activity through a set of germline-encoded receptors and function independently of HLA matching, offering broader applicability and heighted anti-tumor immunity (24). NK cells can be sourced from multiple origins including peripheral (23, 25, 28) and cord (21, 22, 24, 31) blood as well as induced pluripotent stem cells (30, 39–41) and immortalized cell lines (42). Advancements in engineering strategies, such as multicistronic constructs incorporating autocrine IL-15 cytokine support, have significantly enhanced their persistence making them attractive contenders for adoptive cell therapy (21–25, 28, 30, 31, 39–41).

Beyond GvHD, a major challenge in the allogeneic setting is host-mediated rejection of infused immune cells, which has spurred the development of ‘stealth’ engineering strategies to help evade immune recognition and promote persistence. These strategies include β2M knockout to disrupt classical HLA class I expression, overexpression of non-classical HLA molecules such as HLA-E or HLA-G, and the incorporation of alloimmune-defense receptors (ADR) to selectively eliminate alloreactive host immune cells.

**Figure 1:**
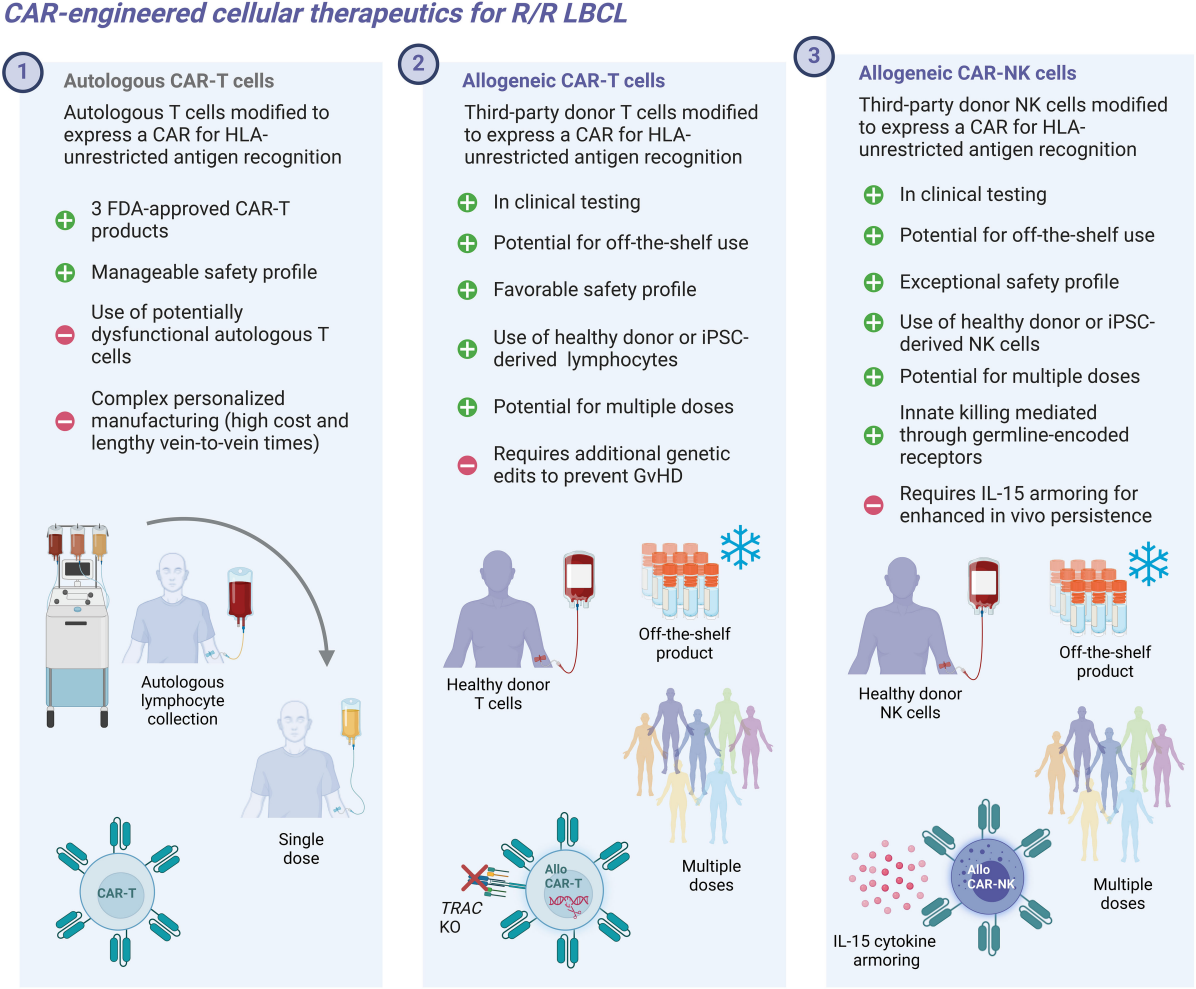
Conceptual depicting characteristics of autologous and allogeneic CAR-engineered cellular therapeutics.

Accelerated by these advances in cellular engineering technology, allogeneic CAR-T and CAR-NK cell therapies have emerged as clinical candidates for r/r large B cell lymphoma (LBCL) (**Figure 1**). Early clinical trial data, although limited by small patient cohorts, provide critical insights into the feasibility, safety, and antitumor efficacy of these novel therapeutic modalities.

This systematic review and meta-analysis is the first to comprehensively evaluate the emerging clinical evidence on the safety and antitumor efficacy of allogeneic CAR-engineered cell products for the treatment of r/r LBCL. By synthesizing early clinical trial data, we aim to provide an up-to-date and comprehensive evaluation of allogeneic CAR-T and CAR-NK cell therapies. These findings will help assess the feasibility and potential therapeutic impact of these next-generation cell therapies to overcome the limitations of autologous CAR-T cells and transform the therapeutic landscape for r/r LBCL.

## Methods

### Data sources, eligibility criteria and search strategy

This systematic review and meta-analysis was conducted in accordance with the PRISMA guidelines (43). MEDLINE, EMBASE, Web of Science, and the Cochrane CENTRAL Register of Controlled Trials were searched for interventional clinical trials, with or without comparator, which investigate allogeneic CAR-engineered adoptive cell therapies in patients with large B cell lymphoma from inception to January 12, 2025. Additionally, the conference proceedings of the American Society of Hematology, the American Society of Clinical Oncology, the American Association for Cancer Research, the American Society of Transplantation and Cellular Therapy, the European Hematology Association, International Conference on Malignant Lymphoma were searched manually. Only studies which reported the primary outcomes overall response rate (ORR), and complete response rate (CRR) were eligible for inclusion. Full-length articles, conference abstracts, letters, and case reports were included, whereas reviews, editorials, and commentaries were excluded. Studies without an identifiable associated clinical trial (verified through a clinical trial registration number) were excluded to prevent double counting of participants. In instances where peer-reviewed data or trial results were unavailable, data from conference proceedings or reports issued by trial sponsors were considered. Such data were included only if they provided sufficient methodological detail and could be corroborated through clinical trial registries. In addition to searching study reports, ClinicalTrials.gov and the WHO International Clinical Trials Registry Platform (ICTRP) were consulted to identify and catalog relevant registered clinical trials.

We searched using MeSH terms and keywords for (“Chimeric antigen receptor” OR “T cells” OR Natural Killer Cells” OR “Adoptive Cell Transfer” OR “Adoptive Immunotherapy” OR “Cell- and Tissue-based therapy”) AND “Allogeneic Cells” AND (B-cell Lymphoma OR Non-Hodgkin Lymphoma” OR “Diffuse Large B-Cell Lymphoma”) from the date of inception to January 12, 2025. No filters or publication time limits were applied for the search. A total of 955 unique records were identified using the database search. No language restrictions were applied. All search results were imported to the Endnote reference manager, and duplicates were removed.

### Study selection

The search results were imported into Covidence systematic review software (Veritas Health Innovation, Melbourne, Australia). Title and abstract screening, full-text screening, data extraction, and risk of bias assessment were performed independently by two reviewers (AB and JSH). Discrepancies were resolved through discussion or by consultation with a third reviewer (FB). The inclusion criteria were as follows: (1) original studies, specifically early-phase clinical trials; (2) studies including adult participants; (3) studies involving patients with relapsed/refractory large B-cell lymphoma (r/r LBCL), including diffuse large B-cell lymphoma (DLBCL) and high-grade B-cell lymphoma (HGBCL); (4) studies evaluating allogeneic CAR-engineered immune effector cell therapy as the intervention; (5) studies including patients regardless of prior exposure to CD19-targeting agents; and (6) studies including patients with or without previous CAR-T cell therapy. Studies requiring matched or partially HLA matched donors (7) as well as studies utilizing TCR-engineered T cell products (8) were excluded from analysis. Studies evaluating allogeneic CAR-T cells derived from a previous allogeneic hematopoietic stem cell transplantation (allo-HSCT) donor (9) were similarly excluded.

For studies with multiple reports, related records were identified and grouped based on their associated clinical trial number. The most recent full-length article was designated as the primary report for data extraction. If no full-length article was available, the most recent conference abstract or sponsor-issued report was used as the primary report.

### Data extraction

Three authors independently extracted data from the 19 selected studies (29, 31–33, 44–58), and the datasheets were cross-checked to resolve any discrepancies. Data collected included available baseline characteristics (e.g., number of patients, diagnosis, prior lines of therapy, prior exposure to CD19-targeting agents, prior CAR-T therapy) as well as primary and secondary outcomes.

The primary outcomes extracted for meta-analysis were the best objective response rate (bORR) and best complete response rate (bCRR), defined as the proportion of patients achieving an objective response (OR) or complete response (CR), respectively, at any time during follow-up.

Response definitions were based on the criteria reported in the respective clinical trials and were not standardized across studies. Cases where response assessments were not explicitly reported were excluded from the analysis.

Secondary efficacy outcomes including overall survival (OS), progression-free survival (PFS), duration of response (DOR), duration of complete remission were extracted when available. Secondary safety outcomes comprised the reported incidence of adverse events, including cytokine release syndrome (CRS), immune-effector cell-associated neurotoxicity syndrome (ICANS), graft-versus-host disease (GVHD), infections, and other reported adverse events. Severity of reported adverse events were recorded and aggregated as low-grade (grade 1-2) or high-grade (grade 3+) toxicities for data analysis purposes.

### Data analysis

Meta-analysis was conducted for the primary response criteria bORR and bCRR for r/r LBCL patients. Safety analysis was performed by evaluating all infused r/r B-NHL study patients (including additional histologies) for the incidence of infections, CRS, ICANS and GvHD. Safety analysis for the incidence of infections, CRS and ICANS were performed independently for low-grade (grade 1-2) and high-grade events (grade 3+). Response and safety assessments were performed in aggregate as well as independently by modality (CAR-NK vs. CAR-T). CAR-NKT cells were grouped among CAR-T cells due to their biological similarity to T cells in terms of lineage and TCR expression.

Analyses were performed using STATA/BE statistical software (v.18.0). Binary outcomes were summarized as proportions with 95% confidence intervals (CI). A random-effects model (Restricted Maximum Likelihood) was applied to pool proportions using an arcsine-based transformation, implemented via the meta set function. Due to the prevalence of zero-event observations, particularly in the safety analysis, arcsine transformation was employed for analysis of pooled estimates. Heterogeneity among studies was assessed using several metrics. Between-study variance (τ (2)) was estimated using a random-effects model. The I² statistic was calculated to quantify the proportion of variability in effect sizes due to heterogeneity. Cochran’s *Q*-test was performed to evaluate the significance of heterogeneity, with the null hypothesis of no heterogeneity tested at a significance level of 0.05. Subgroup analyses were conducted using the subgroup function in STATA/BE, focusing on pre-specified subgroups: CAR-T cells versus CAR-NK cells. Forest plots were created using Graphpad PRISM (Version 10.4.1 Build 532).

## Results

### Search results

**Figure 2:**
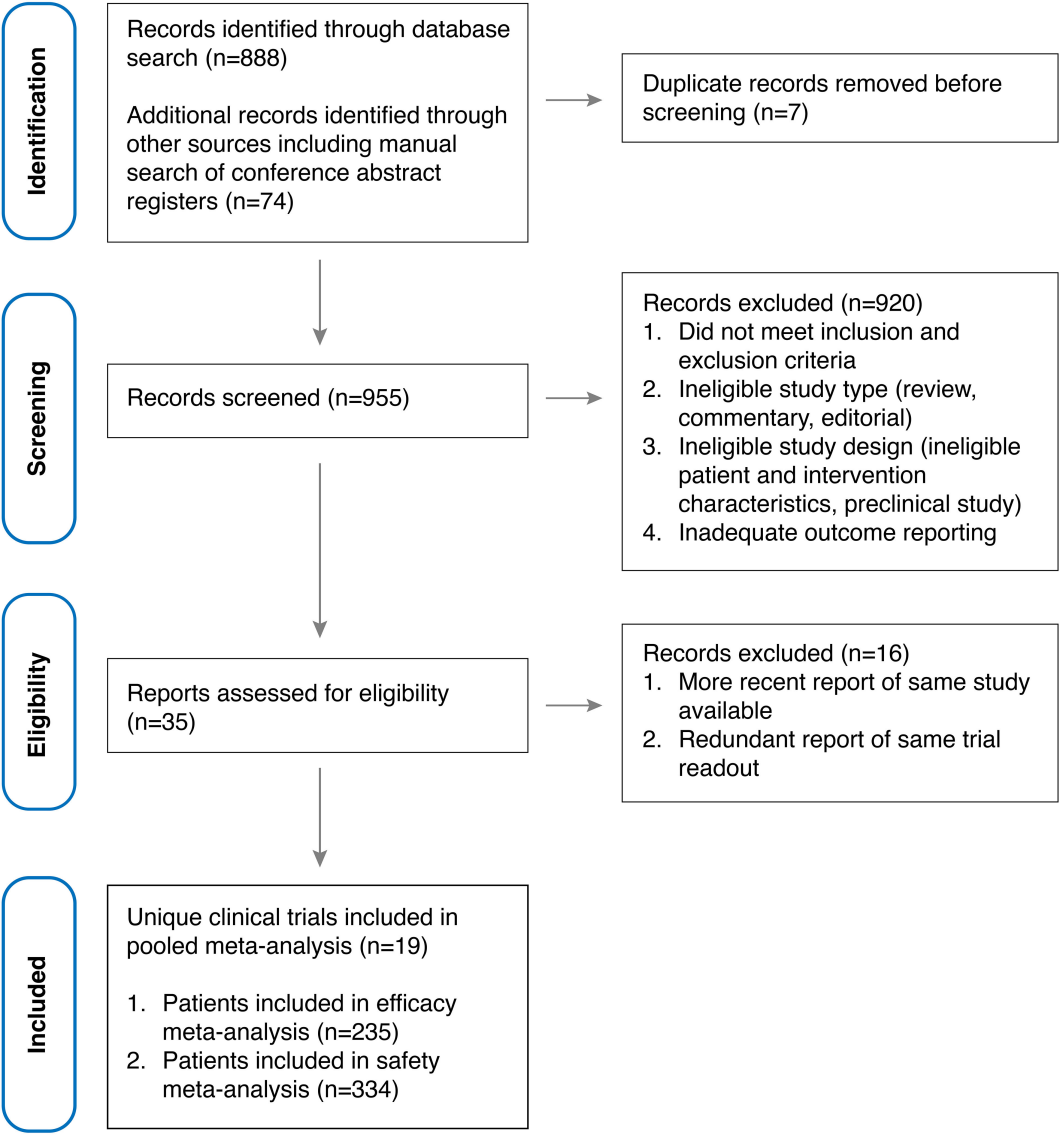
PRISMA flow diagram illustrating the identification, screening, exclusion, and inclusion of clinical trials.

Out of 953 unique references identified, 35 reports (22, 23, 35, 36, 39–41, 44–61) of early-phase clinical trials investigating allogeneic CAR-transduced immune effector cells (CAR-IEC) for the treatment of relapsed and refractory large B cell lymphoma (r/r LBCL) were screened for our review. Among these, 19 unique studies (29, 31–33, 44–58) were included in the meta-analysis, encompassing 235 patients evaluable for efficacy analysis and 334 patients evaluable for safety analysis (**Figure 2**). Notably, one study (NCT04030195 investigating PBCAR20A) (62) was excluded from both the efficacy and safety meta-analyses due to inconsistent data reporting; however, it is included in the tabular data synthesis. Included studies included phase 1, phase 2 and phase 1/2 early clinical trials, most of which were single-arm, open-label non-randomized trials. Geographically, studies were predominantly sponsored by US-based development programs (11/19) with three trials each sponsored out of Europe and China, respectively, one out of Australia and another out of Japan. Investigational CAR-IEC development programs were mostly industry-sponsored (17/19) with only two investigator-initiated studies sponsored by academic institutions (**Supplementary Figure S1**). Detailed characteristics of the included studies and interventions are provided in **Table 1**, while response and safety data are summarized in **Tables 2**, **3**, respectively.

**Table 1:** Patient and intervention characteristics for studies evaluating allogeneic CD19-CAR-engineered cellular therapeutics in large B cell lymphoma (LBCL).

*Allogeneic CD19-CAR cell products*	Trial details & identifier	Sponsor	Key inclusion and exclusion criteria & patient characteristics	Cell source	Genetic edits and rational	Cell dose and schedule
PBCAR19B	NCT04649112	Precision BioSciences	• R/R B-NHL • min. of ≥ 2 PLoT • Prior HSCT and autoCAR-T eligible • Median age: 67	T cells (PBMCs)	• CD19-CAR targeted to *TRAC* locus by ARCUS nuclease replacing endogenous TCR to prevent GvHD • Anti-B2M shRNA to reduce MHC 1 expression to prevent rejection by T cells • HLA-E transgene to prevent rejection by NK cells	Standard LD: fludarabine 30 mg/m2/d × 3d + cyclophosphamide 500 mg/m2/d × 3d Augmented cyclophosphamide (Aug-Cy) LD: 750 mg/m2/d × 3d + fludarabine 30 mg/m2/d × 3d Single CAR-T infusion (flat dose) of 270M (DL1) to 540M (DL2)
FT522	Phase 1 multicenter trial (NCT05950334)	Fate Therapeutics	• R/R B-NHL • min. of ≥ 1 PLoT • No prior alloHSCT or alloCAR-T therapy within 6 months of Day 1	NK cells (iPSCs)	• CD19-CAR CD3ζ • High affinity, non-cleavable CD16 Fc receptor (hnCD16) • CD38 KO to prevent NK cell fratricide, enhance metabolic fitness • IL-15/IL-15 receptor fusion • Alloimmune-defense receptor (ADR) targeting 4-1BB	Regimen A: • Fludarabine (30mg/m^2^)/Cyclophosphamide (500mg/m^2^) (d-5 to -3) • Single-dose Rituximab (d-4) • 3 CAR-NK cell infusions (d1, 4 and 8) • DL1 – 3x300M cells/dose (up to 2 cycles) • DL2: 900M cells/dose
FT596	Phase 1 multicenter trial (NCT04245722)	Fate Therapeutics	• R/R B-NHL (n=68) • Min. of ≥ 1 PLoT • No prior alloHSCT or allogeneic CAR-T within 6 months of d1 • Median of 4 prior lines of therapy • 84% Stage IV disease • R/R LBCL (n=32)	NK cells (iPSCs)	• CD19-CAR • High affinity, non-cleavable CD16 Fc receptor (hnCD16) • IL-15/IL-15 receptor fusion	Regimen A • Fludarabine (30mg/m^2^)/Cyclophosphamide (500mg/m^2^) (d-5 to -3) • Single-dose Rituximab (d-4) • Up to 3 CAR-NK cell infusions (d1, 4 and 8) • Dose levels from 30M to 1.8bn cells/dose
FT819-101	Phase I dose-finding study (NCT04629729)	Fate Therapeutics	• R/R B-NHL • min. of ≥ 2 PLoT • Prior CD19-targeted CAR T-cell therapy permitted • Documented surface CD19 expression after relapse	T cells (iPSCs)	• 1XX CAR19 with CD28 costimulatory domain and modified CD3ζ targeted to the *TRAC* locus • Biallelic disruption of *TRAC* at clonal level to delete endogenous TCR to prevent GvHD • CD58 KO • Alloimmune-defense receptor (ADR) targeting 4-1BB	• LD with fludarabine (30 mg/m2)/cyclophosphamide (500 mg/m2) on d-5 to -3 • Single (d1) or fractioned (d1, 3, and 5) doses of 30-900M cells of FT819 as monotherapy or in combination with IL-2
NKX019-101	Open label, multicenter Phase 1 trial (NCT05020678)	Nkarta	• R/R B-NHL (n=19) • min. of ≥ 2 PLoT • Median (range) PLoT: 4 (2-10) • No prior autologous CD19 CAR-T exposure • No prior alloHSCT	NK cells (PBMCs)	• Humanized CD19(FMC63 scvs).OX40.CD3ζ CAR • Membrane-bound IL-15 for autocrine growth support and enhanced persistence	• LD (fludarabine (30mg/m^2^/d)/cyclophosphamide (300mg/m2/d)) followed by 3 doses of NKX019 on d0, 7, and 14 (after protocol amendment: d0, 3, and 7) • Dose escalation: 300 x 10^8^ to 2 x 10^9^ CAR NK+ cells/dose • Subjects could receive additional treatment cycles to deepen response, for remission consolidation, as well as for retreatment of initial CR and subsequent relapse.
CAR19/IL15 NK	NCT03056339	The University of Texas M.D. Anderson Cancer Center	• R/R B-NHL(n=37) • Median age (range): 64 (26-79) • min. of ≥ 2 PLoT • Median (range) number of prior therapies: 4 (2-10) • Stage III/IV disease: 14 (63.6%) • R/R DLBCL (n=17) • No prior CD19-CAR-T (for dose extension phase) • Prior alloHSCT permitted	NK cells (CBMCs)	• CD19-CAR.CD28.CD3ζ • Autocrine IL-15 armoring for enhanced *in vivo* persistence • Inducible Caspase9 safety switch	Cyclophosphamide (300mg/m^2^) (d-5 to -3) Single CAR-NK infusion (d0) Dose escalation (n=11): Dose level 1: 0.1M cells/kg (n=3) Dose level 2: 1M cells/kg (n=4) Dose level 3: 10M cells/kg (n=4) Dose expansion (n=26): 10M cells/kg (n=11) Flat dose 800M cells (n=15)
CTX110	Phase 1 CARBON open-label, multicenter, study (NCT04035434)	CRISPR Therapeutics	• R/R B-NHL • min. of ≥ 2 PLoT • No prior allogeneic HSCT or CAR-T therapy	T cells (PBMCs)	• CD19 CAR • TCR disruption to prevent GvHD • B2M KO to eliminate MHC1 expression	• LD with fludarabine (30mg/m^2^/d) and cyclophosphamide (500mg/m^2^/d) • Single infusion of 30-600M cells, redosing allowed for relapse
CTX112	Open-label, multicenter, Phase 1/2 study (NCT05643742)	CRISPR Therapeutics	• R/R B-NHL • No prior allogeneic HSCT • min. of ≥ 2 PLoT • Prior CD19 autologous CAR-T eligible • Median age: 62 (49-79) • Stage IV disease: 66.7% • LDH>ULN: 50% • Median PLoT: 3 (1-7) • Primary refractory disease: 58%	T cells (PBMCs)	• CD19 CAR KI into *TRAC* locus using an AAV template • TCR disruption to prevent GvHD • *B2M* KO to eliminate MHC1 expression • *TGFBR2* KO to overcome immunosuppression in the TME • *YC3H12A* (Regnase-1) to increase cell expansion and functional persistence	LD with fludarabine 30 mg/m^2^ and cyclophosphamide 500 mg/m^2^ for 3 days Single infusion of 30-600M cells
RJMty19	Open-label, single-dose, phase 1 study (NCT05453669)	Guangdong Ruishun Biotech Co., Ltd	• R/R high-grade B-NHL • min. of ≥ 2 PLoT • No prior CAR-T therapy	Double-negative T cells (PBMCs)	• Humanized CD19(scFv).4-1BB.CD3ζ CAR	LD with fludarabine 30 mg/m^2^ and cyclophosphamide 500 mg/m^2^ for 3 days Single infusion of 1-20M cells/kg body weight on d0
ALLO-501/Cema-cel (ALLO-501A)	2 multicenter, single-arm, open-label, phase 1 trials (ALPHA: NCT03939026 & ALPHA2: NCT04416984	Allogene Therapeutics	• R/R LBCL • Autologous CAR T-naïve pts • min. of ≥ 2 PLoT • Median PLoT: 3 • Prior transplant: 50% • 67% Stage IV disease • Median age: 60y • Baseline LDH>ULN: 67% • IPI 3-5: 50% • GCB subtype (50%) • Extranodal disease (58%)	T cells (PBMCs)	• CD19(scFv).4-1BB.CD3ζ CAR • TCR KO to prevent GvHD (utilizing TALEN gene editing) • CD52 KO to enable administration of anti-CD52 lymphodepleting antibodies (utilizing TALEN gene editing) • ALLO-501 only: Rituximab recognition domain as kill switch	• Fludarabine (30 mg/m^2^/day), cyclophosphamide (300 mg/m^2^/day), ALLO-647 ([anti-CD 52 mAb] 30 mg/day; total dose: 90 mg) (d-5 to -3) • Single dose of 120M (DL2) ALLO-501 or ALLO-501A (d0)
Azer-cel (PBCAR0191)	Phase 1/2a, nonrandomized, open-label, parallel assignment, dose-escalation, and dose-expansion study (NCT03666000)	Imugene Limited	• R/R B-NHL • Min. of ≥ 2 PLoT • ≤ 7 PLoT • Prior CD19 autologous CAR-T eligible	T cells (PBMCs)	• CD19 CAR targeted to the *TRAC* locus utilizing ARCUS gene editing	• LD with fludarabine/cyclophosphamide • Infusion of 0.3M to 9M cells/kg body weight in one single infusion (DL1,2,3a) or split into 2 (DL4)/3 (DLs 3b & DL5) infusions
CB-010	ANTLER Phase 1 multicenter, open-label dose escalation and dose expansion study (NCT04637763)	Caribou Biosciences	• R/R B-NHL • Min. of ≥ 2 PLoT or primary refractory disease to 1L therapy • No prior therapy with CD19-targeting agent • No prior alloHSCT • Median PLoT (range): 1 (1-8) • Median age (range): 65 (21-82) • IPI≥3: 39.1% • LDH>ULN: 50%	T cells (PBMCs)	• CD19-CAR (scFv FMC63) targeted to *TRAC* locus replacing endogenous TCR to prevent GvHD • PD-1 KO to prevent premature CAR-T cell exhaustion and potentially enhance antitumor activity • CRISPR hybrid RNA-DNA (chRDNA) technology	• LD with cyclophosphamide (60 mg/kg/day x 2 days) and fludarabine (25 mg/m2/day x 5 days) • Single dose of 40-120M cells (flat dose)
ADI-001	Open-label, multi-center phase 1 GLEAN trial (NCT04735471)	Adicet Bio	• High grade R/R B-NHL • Min. of ≥ 2 PLoT • Prior CD19 autologous CAR-T eligible (n=12, 50%) • Median age: 66.5y (range 44-75) • Median IPI score (LBCL): 2.5 (1-4) • Stage IV disease: 17 (70.8%) • Median PLoT: 4 (range 2-9)	γδ T cells (PBMCs)	• CD20 CAR • No gene editing	Standard LD (sLD): Cyclophosphamide 500 mg/m2 (3d) and fludarabine 30 mg/m2 (d) Enhanced LD (eLD): Cyclophosphamide 1000 mg/m2 (3d) and fludarabine 30 mg/m2 (4d) 1 (to 2) infusion(s) on d1 (+d7) of 30M-1bn cells
U-CAR-T19	Phase 1 clinical trial (NCT03229876)	Bioray Laboratories	• R/R B/ALL and R/R B-NHL • Age range R/R B-NHL: 18-65y • PLoT range (2-4) • Prior CD19 autologous CAR-T eligible • No prior alloHSCT	T cells (PBMCs)	• CD19(scFv FMC63).4-1BB.CD3ζ CAR • *TRAC* KO to prevent GvHD • *B2M* KO to disrupt MHC1 and prevent rejection by alloreactive T cells	• LD with fludarabine (25 mg/m^2^, D-6 to D-4), cyclophosphamide (500 mg/m^2^, D-6 to D-5) • Cell dose of 1–6M/kg split into two infusions (d0)
UCART20x22	Phase 1/2a NatHaLi-01 clinical trial (NCT05607420)	Cellectis	• R/R B-NHL • Min. of ≥ 2 PLoT • Prior CD19 autologous CAR-T eligible (n=2/3, 66.7%) • # of PLoT: 3-10	T cells (PBMCs)	• CD20 CAR + CD22 CAR • *TRAC* KO to prevent GvHD • CD52 KO to enable use of anti-CD52 mAbs • TALEN gene editing	• LD with fludarabine 30 mg/m ^2^ × 3d, cyclophosphamide 500mg/m ^2^ × 3d, CLLS52–12 mg on D1, 24 mg on D2, D3) • Single infusion of 50M-450M ells at a flat dose level
ET-901 (ATHENA CAR T)	Single center phase 1/2 dose escalation study (NCT06014073)	Chinese PLA General Hospital	• R/R B-NHL • No prior allogeneic HSCT or CAR-T therapy • Median # PLoT: 5	T cells (PBMCs)	• CD19 (scFv FMC63).CD28. CD3ζ CAR • *TRAC* KO to prevent GvHD • Proprietary ‘GeneX’ KO for enhanced T cell persistence	• LD with fludarabine (30–50 mg/m^2^/d) and cyclophosphamide (500–1000 mg/m^2^/d), • Single infusion 1-10M cells/kg body weight
KUR-502	Phase 1 ANCHOR study (NCT03774654)	Athenex, Inc. (filed Chapter 11)	• R/R B-NHL and R/R/B-ALL • Min. of ≥ 2 PLoT • Prior CD19 autologous CAR-T eligible	NKT cells (PBMCs)	• CD19 CAR • IL-15 • shRNA to downregulate B2M and CD74	• LD fludarabine/cyclophosphamide • Single infusion of 10-30M CAR-NKT cells/m2 body surface
SC291	Phase 1 ARDENT trial (NCT05878184)	Sana Biotechnology	• R/R B-NHL • Min. of ≥ 2 PLoT • No prior anti-CD19 agents; dose expansion only: prior approved CD19-directed CAR T therapy required • No alloHSCT permitted	T cells (PBMCs)	• CD19 CAR • TCR disruption • Disruption of MHC class I and II for host immune evasion • CD47 overexpression to evade innate immune recognition via Crispr-Cas12b editing	• LD with fludarabine/cyclophosphamide • Single infusion of 60-120M CAR-T cells
TAK-007	Phase 2 trial (NCT05020015)	Takeda	• R/R B-NHL • Min. of ≥ 2 PLoT • Prior alloHSCT/CAR-T permitted (if>3mo before enrolment) • Median # PLoT: 5 (range, 2-11) • Prior CD19 CAR-T exposure (11/26, 42%) • Outpatient administration: 50%	NK cells (CBMCs)	• iCD19.CD28. CD3ζ CAR • Inducible Caspase9 safety switch • IL-15 cytokine support	• LD fludarabine (30 mg/m^2^) and cyclophosphamide (300 mg/m^2^) • Single or three infusions of 200M (DL1) or 800M (DL2) CAR-NK cells on d0, 7, and 14

Azer-cel, Azercabtagene zapreleucel; Cema-cel, Cemacabtagene ansegedleucel; CRS, cytokine release syndrome; DLBCL, diffuse large B cell lymphoma; GvHD, graft-versus-host disease; HSCT, hematopoietic stem cell transplant; ICANS, immune effector cell neurotoxicity syndrome; LD, lymphodepletion; mAb, monoclonal anitobody; nd, not disclosed; PLoT, prior lines of therapy.

**Table 2:** Efficacy data of allogeneic CD19-CAR-engineered cellular therapeutics in large B cell lymphoma (LBCL).

Allogeneic CD19-CAR cell products	Modality	Evaluable LBCL patients for efficacy *n*	Overall response *n* (%)	Complete response *n* (%)	Duration of response data
FT522	CAR-NK	5	2 (40.0)	1 (20.0)	nd
FT596	CAR-NK	32	12 (37.5)	8 (25.0)	Median DOR not reached at median follow-up time of 9.1m
NKX019-101	CAR-NK	6	2 (33.3)	2 (33.3)	2 (33%) R/R LBCL pts sustained CR min. >4m including 1 (16.7%) pt with ongoing DOR >10m
CAR19/IL15 NK	CAR-NK	17	7 (41.2)	5 (29.4)	1-year PFS: 29.4 (13.2-53.2) 1-year OS: 64.7 (41.3–82.7)
FT819-101	CAR-T	10	3 (30.0)	2 (20.0)	Median (range) DOR: 52d (22-163) 2 responses ongoing at d25 and d163, respectively
Azer-cel (PBCAR0191)	CAR-T	9	4 (44.4)	3 (33.3)	Cohort A: DOR <60d Cohort B: DOR >120d & >90d; all patients ongoing
PBCAR19B	CAR-T	8	6 (75.0)	5 (62.5)	1 CR sustained for 6m. 4 CRs sustained for 3-12+ months.
CTX110	CAR-T	32	18 (56.3)	11 (34.6)	6-mo CR rate for ≥DL3: 19% (5/27)
CTX112	CAR-T	5	2 (40.0)	1 (20.0)	6-mo CR rate (LBCL): 0%
RJMty19	CAR-T	12	4 (33.3)	1 (8.3)	2 responses ongoing for 150d
ALLO-501/ Cema-cel (ALLO-501A)	CAR-T	12	8 (66.7)	7 (58.3)	Median DOR: 23.1m Among 8 pts with 6m follow-up, 5 (62.5%) achieved CR and 4 (50.0%) sustained CR through 6m
CB-010	CAR-T	40	29 (72.5)	17 (42.5)	6-mo PFS: 28% Median (range) duration of CR: 7mo (1-23+) Median PFS (range): 3mo (1-24+)
ADI-001	CAR-T	18	12 (66.7)	11 (61.1)	6m-CR rate: 4/18 (22.2%)
U-CAR-T19	CAR-T	3	0 (0)	0 (0)	No clinical responses due to lack of CAR-T expansion
UCART20x22	CAR-T	3	3 (100)	2 (66.7)	No DOR beyond d28 disclosed
ET-901 (ATHENA CAR T)	CAR-T	4	4 (100)	nd*	nd
TAK-007	CAR-NK	17	7 (41.2)	3 (17.6)	Median DOR (LBCL): 3.4mo
PBCAR20A	CAR-T	17	nd*	nd*	Nd*

*No LBCL-specific response data was reported. Azer-cel, Azercabtagene zapreleucel; Cema-cel, Cemacabtagene ansegedleucel; CR, complete response; CRS, cytokine release syndrome; DLBCL, diffuse large B cell lymphoma; DOR, duration of response; GvHD, graft-versus-host disease; ICANS, immune effector cell neurotoxicity syndrome; PFS, progression-free survival; nd, not disclosed; R/R LBCL, relapsed and refractory large B cell lymphoma; *Trial excluded due to inadequate outcome reporting.

**Table 3:** Safety data of allogeneic CD19-CAR-engineered cellular therapeutics in large B cell lymphoma (LBCL).

Allogeneic CD19-CAR cell products	Evaluable patients for safety	CRS grade 1-2 *n* (%)		CRS grade 3+ *n* (%)	ICANS grade 1-2 *n* (%)		ICANS grade 3+ *n* (%)	Infections grade 1-2 *n* (%)		Infections grade 3+ *n* (%)	GvHD *n* (%)	Dose-limiting toxicities *n* (%)
FT522	5 (R/R LBCL) 3 (R/R indolent B-NHL) 1 (R/R MCL)	0 (0)		0 (0)	0 (0)		0 (0)	nd		nd	0 (0)	0 (0)
FT596	68 (R/R B-NHL)	10 (14.0)		0 (0)	0 (0)		0 (0)	21 (30.9)		11 (16.2)	0 (0)	0 (0)
NKX019-101	19 (R/R B-NHL)	5 (26.3)		0 (0)	0 (0)		0 (0)	nd		1 (5.3)	0 (0)	0 (0)
CAR19/IL15 NK	37 (R/R B-NHL)	1 (2.7)		0 (0)	0 (0)		0 (0)	7 (18.0)		4 (10.8)	0 (0)	0 (0)
FT819-101	10 (R/R LBCL)	1 (10.0)		0 (0)	0 (0)		0 (0)	nd		0 (0)	0 (0)	0 (0)
Azer-cel (PBCAR0191)	9 (R/R LBCL)	4 (44.4)		0 (0)	0 (0)		2 (22.2)	5 (55.6)		2 (22.2)	0 (0)	nd
PBCAR19B	5 (R/R LBCL)	nd		0 (0)	nd		0 (0)	nd		0 (0)	0 (0)	nd
CTX110	32 (R/R LBCL)	18 (56.3)		0 (0)	1 (3.1)		2 (33.3)	4 (12.5)		4 (12.5)	0 (0)	1 death due to HHV6 encephalitis
CTX112	12 (R/R B-NHL)	7 (58.3)		0 (0)	4 (33.3)		0 (0)	5 (41.7)		0 (0)	0 (0)	0 (0)
RJMty19	12 (R/R LBCL)	4 (33.3)		0 (0)	0 (0)		0 (0)	1 (8.3)		0 (0)	0 (0)	0 (0)
ALLO-501/Cema-cel (ALLO-501A)	12 (R/R LBCL)	4 (33.3)		0 (0)	0 (0)		0 (0)	8 (66.7)		1 (8.3)	0 (0)	0 (0)
CB-010	40 (R/R LBCL)	23 (57.5)		0 (0)	6 (15.0)		2 (5)	11 (27.5)		8 (20.0)	0 (0)	1 death possibly related to CB-010 per investigator due to complications of a bladder perforation in the context of BK virus hemorrhagic cystitis
ADI-001	24 (R/R B-NHL)	10 (41.7)		1 (4.2)	2 (8.3)		1 (4.2)	5 (20.8)		6 (25.0)	0 (0)	0 (0)
U-CAR-T19	3 (R/R/LBCL)	3 (100.0)		0 (0)	0 (0)		0 (0)	0 (0)		0 (0)	0 (0)	0 (0)
UCART20x22	3 (R/R LBCL)	3 (100.0)		0 (0)	0 (0)		0 (0)	nd		1 (33.3)	0 (0)	0 (0)
TAK-007	26 (R/R B-NHL)	3 (11.5)		0 (0)	0 (0)		0 (0)	nd		nd	0 (0)	0 (0)
ET-901 (ATHENA CAR T)	6 (R/R B-NHL)	nd, 6 (100)*			nd, 2 (33.3)*			nd, 2 (33.3)*			1 (16.7)	0 (0)
KUR-502	5 (R/R B-NHL)	0 (0)	0 (0)		0 (0)	0 (0)		nd	0 (0)		0 (0)	0 (0)
PBCAR20A*	17 (R/R B-NHL or R/R CLL/SLL)	nd* 5 (29.4)			nd* 1 (5.9)			Nd**			nd	1 (5.9%)

*Number of any grade adverse event is depicted due to lack of granular safety data. **Safety data not reported with patient-level granularity. Azer-cel, Azercabtagene zapreleucel; Cema-cel, Cemacabtagene ansegedleucel; CRS, cytokine release syndrome; DLBCL, diffuse large B cell lymphoma; GvHD, graft-versus-host disease; ICANS, immune effector cell neurotoxicity syndrome; nd, not disclosed; * Trial excluded due to inadequate outcome reporting.

### Patient characteristics

All included studies reported response data for r/r LBCL patients, while a significant subset of studies (9/19) also included R/R B-NHL patients with additional histologies encompassing follicular lymphoma (FL), primary mediastinal B cell lymphoma (PMBCL), marginal zone lymphoma (MZL), or mantle cell lymphoma (MCL), which represented the basis for the pooled safety analysis of this review. Of all included studies, 10/19 allowed prior allogeneic hematopoietic cell transplant and 11/19 permitted prior exposure to autologous CD19-CAR-T cell therapy with varying minimum wash-out periods before infusion of investigational allogeneic cell products. Where available, the fraction of trial subjects who previously received autologous CAR-T therapy is specified in **Table 1** as well as other information on patient demographics, disease status, and prior lines of therapy. The majority of studies (16/19) investigated patients who had received a minimum of 2 prior lines of therapy (PLoT) while 3 out of 19 studies investigated patients who had received a minimum of 1 prior line of therapy. Included studies encompassed a median of 12 (range 3-68) infused patients.

### Intervention characteristics

Included studies investigated CAR-NK (5/19), CAR-T (13/19) and CAR-NKT (1/19) investigational cell products targeting CD19 (17/19), CD20 (1/19) or CD20/CD22 (1/19).

For CAR-NK cell products, cell sources included peripheral (1/5) and cord blood (2/5) as well as induced pluripotent stem cells (2/5). CAR-T cells were manufactured from peripheral blood mononuclear cells (13/14) or induced pluripotent stem cells (1/14). T cell employed for cellular engineering were mostly αβ T cells (13/14) including one study with double-negative CD4^-^/CD8^-^ αβ T cells (1/14), while one study leveraged γΔ T cells (1/14). Investigational cell products harbored a variety of additional genetic modifications or molecular payloads for enhanced functionality or increased safety leveraging a multitude of gene editing platform including TALEN (2/19), ARCUS (2/19) and CRISPR (7/19) (**Table 1**). All allogeneic CAR-NK cell candidates (5/5) relied on autocrine IL-15 growth support for enhanced *in vivo* persistence in form of an IL-15/IL-15 fusion receptor (2/5), membrane-bound IL-15 (1/5) or autocrine secretion of IL-15 (2/5). CAR-NK cell candidates sourced from iPSCs (2/5) were equipped with a high affinity, non-cleavable CD16 Fc receptor (hnCD16) for antibody-dependent cellular toxicity (ADCC). One CAR-NK cell candidate, FT522, included additional gene edits including CD38 disruption to prevent NK cell fratricide as well as a proprietary alloimmune-defense receptor (ADR) targeting 4-1BB to prevent host rejection (1/5). Of note, none of the included CAR-NK cell products required any genetic modifications to prevent GvHD due to the inherent safety profile of NK cells.

CAR-T cell products, on the other hand, mostly relied on TCR disruption to prevent GvHD using CRISPR (7/14), TALEN (2/14) or ARCUS (2/14) gene editing technology with the exception of three studies which employed double-negative αβ T cells, γΔ T cells and NKT cells, respectively, which lack the propensity to induce GvHD. Strategies employed to reduce host rejection of infused cells included anti-B2M (beta 2 microglobulin) shRNA or B2M KO to reduce MHC-1 expression, ectopic expression of HLA-E to prevent host NK cell-mediated rejection, CD47 overexpression to evade innate immune cell rejection, or insertion of an alloimmune-defense receptor (ADR) targeting 4-1BB. Additional genetic edits to enhance functionality, shield from immunosuppressive pressures and augment anti-tumor potency included CD52 KO to enable administration of anti-CD52 lymphodepleting antibodies, PD-1 KO to prevent premature CAR-T cell exhaustion, *TGFBR2* KO to overcome TME-induced immunosuppression, Regnase-1 KO for enhanced functional persistence and CD74 KO to prevent formation of immunosuppressive Tregs. Safety switches were present in three out of 19 investigational cell products in form of inducible Caspase9 (2/19) or a rituximab recognition domain (1/19). Of note, there were no reported cases in which safety kill switches were employed to mitigate uncontrollable toxicity.

There was inconsistent reporting on costimulatory domains employed to enhance CAR signaling with only 7/19 studies providing detailed information on CAR design. Costimulatory domains included CD28 (4), OX40 (1) and 4-1BB (2) (**Table 1**).

Lymphodepletion regimens administered prior to cell infusion consisted of fludarabine (25-30mg/m^2^) and cyclophosphamide (300-750mg/m^2^) administration on days d-5 to d-3. Infused cell doses ranged from 2x10^7^ to 2x10^9^ and 8/19 studies allowed infusion of multiple dose, three of which were prespecified split/fractioned doses.

Notably, multiple CAR-T/CAR-NK candidates (CTX110, ALLO-501 and FT596), have already been phased out by their respective sponsors to focus their development programs on next-generation candidates (CTX112, Cema-Cel, previously known as ALLO-501A and FT522), which feature improved functionality through additional genetic modifications. Specifically, CTX112 leverages AAV-template directed site-specific CAR insertion and contains additional genetic edits including KO of Regnase-1 to increase functional persistence, cytokine secretion, KO of *TGFBR2* to shield from TME-associated immunosuppression and KO of MHC class 1 for reduced immunogenicity. Cema-Cel (ALLO-501A) lacks the rituximab kill switch present in ALLO-501 to expand the potential patient eligible for treatment including patients with recent exposure to rituximab. Cema-Cel (ALLO-501A) is currently being investigated in the pivotal phase 2 ALPHA3 trial in a first line (1L) LBCL indication to boost first-line cure rates as consolidation after induction therapy. FT522 features an alloimmune-defense receptor (ADR) targeting 4-1BB to reduce alloreactive rejection of infused cells and lacks CD38 to prevent NK cell fratricide and enhance metabolic fitness. Together, these three cell candidate iterations highlight the continuous innovation driven by precision gene editing which allows to custom-build potent cellular therapeutics with advanced functionality for enhanced anti-tumor efficacy.

### Response data

**Figure 3:**
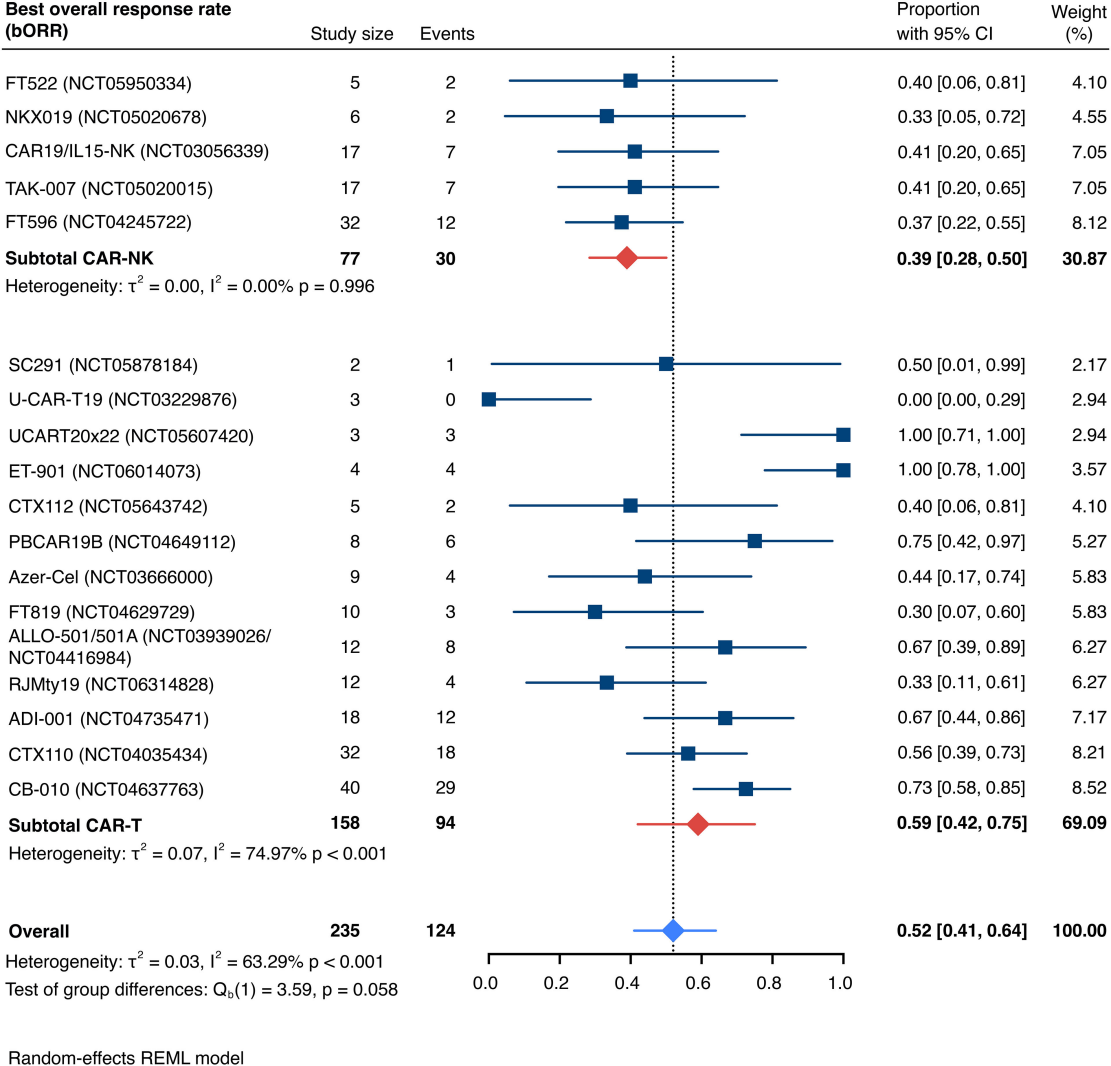
Forest plot illustrating the best overall response rate (bORR), stratified by cell source. Pooled estimates were computed for each subgroup and the overall weighted effect.

This systematic review and meta-analysis included 19 studies (29, 31–33, 44–58) encompassing a total of 235 patients (77 CAR-NK; 158 CAR-T) evaluable for response. The pooled estimate for the best overall response rate (bORR) was 52.5% [95% CI, 41.0-63.9] (n=235) with no significant group-to-group difference (Q_b_(1)=3.59, p=0.058) between investigated CAR-NK or CAR-T cell products (**Figure 3**). The random-effects maximum likelihood (REML) model displayed moderate overall heterogeneity (I^2^ = 63.29%; p<0.001), which was mainly driven by significant heterogeneity among the included CAR-T cell studies (I^2^ = 74.97%, p<0.001). Included CAR-NK cell trials exhibited considerably less heterogeneity (I^2^ = 0.00%, p=0.996) in the all-study model.

**Figure 4:**
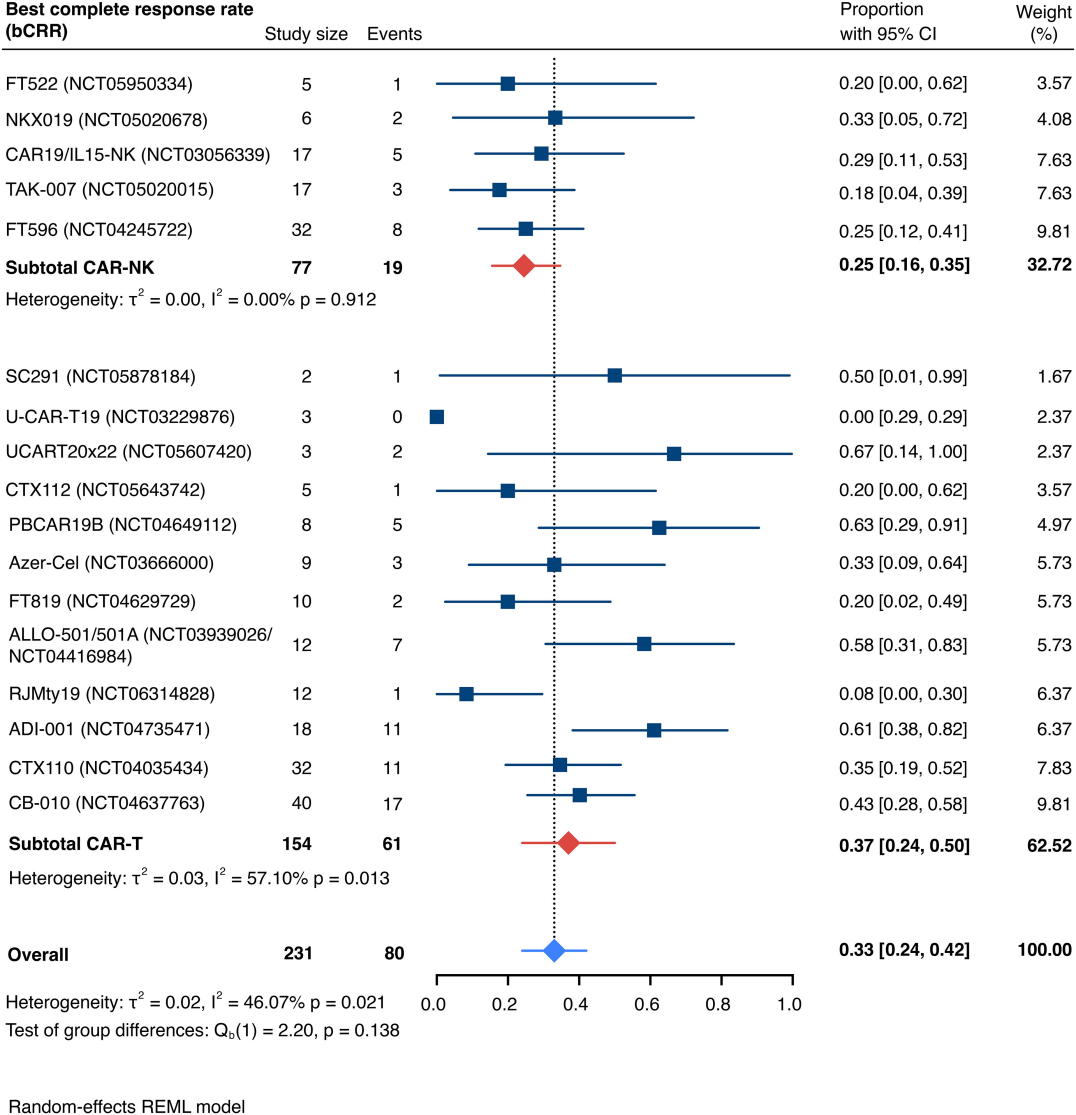
Forest plot illustrating the best complete response rate (bCRR), stratified by cell source. Pooled estimates were computed for each subgroup and the overall weighted effect.

The pooled estimate for the best complete response rate (bCRR) was 32.8% [95% CI, 24.2-42.0] (n=231; **Figure 4**). Similarly, there was no significant superiority of any cell type (Q_b_(1)=2.20, p=0.138). We found moderate overall heterogeneity, exclusively driven by moderate intra-group heterogeneity for included CAR-T cell trials (I^2^ overall=46.07%, p=0.021; I^2^ CAR-NK<0.01%, p=0.912; I^2^ CAR-T=57.10%, p=0.013). Patient-level data were insufficient to conduct subgroup analyses based on age, disease stage, international prognostic index (IPI), number of prior lines of therapy, prior hematopoietic stem cell transplantation (HSCT), previous CD19 CAR T-cell therapy, or other prior immunotherapies.

There was inconsistent reporting on secondary efficacy endpoints including OS, PFS, the duration of response (DOR) and median follow-up across the trials due to the early-stage nature of the clinical data hindering direct comparisons across studies. This variability reflects the limited maturity of outcomes and differing methodologies among early-phase studies. Of note, one trial of CD19-targeted CAR-NK cells reported a 1-year progression free survival rate of 29.4% (13.2-53.2) and a 1-year overall survival rate of 64.7% (41.3-82.7), grossly in line with recent real-world data from autologous CD19-directed CAR-T cell products despite enrolling later-line patients (median # PLoT: 4 [range 3-10] vs. 3 [range 2.6-3.5]) (8). Another study of CD19-targeted CAR-NK cells reported that median duration of response was not reached at a median follow-up of 9.1 months, whereas a study investigating CD19-directed CAR-T cells observed a median duration of response of 23.1 months, indicating the encouraging potential for durable responses with allogeneic CAR-engineered cell products. **Table 2** summarizes data on duration of response and follow-up duration, where available.

### Safety data

**Figure 5:**
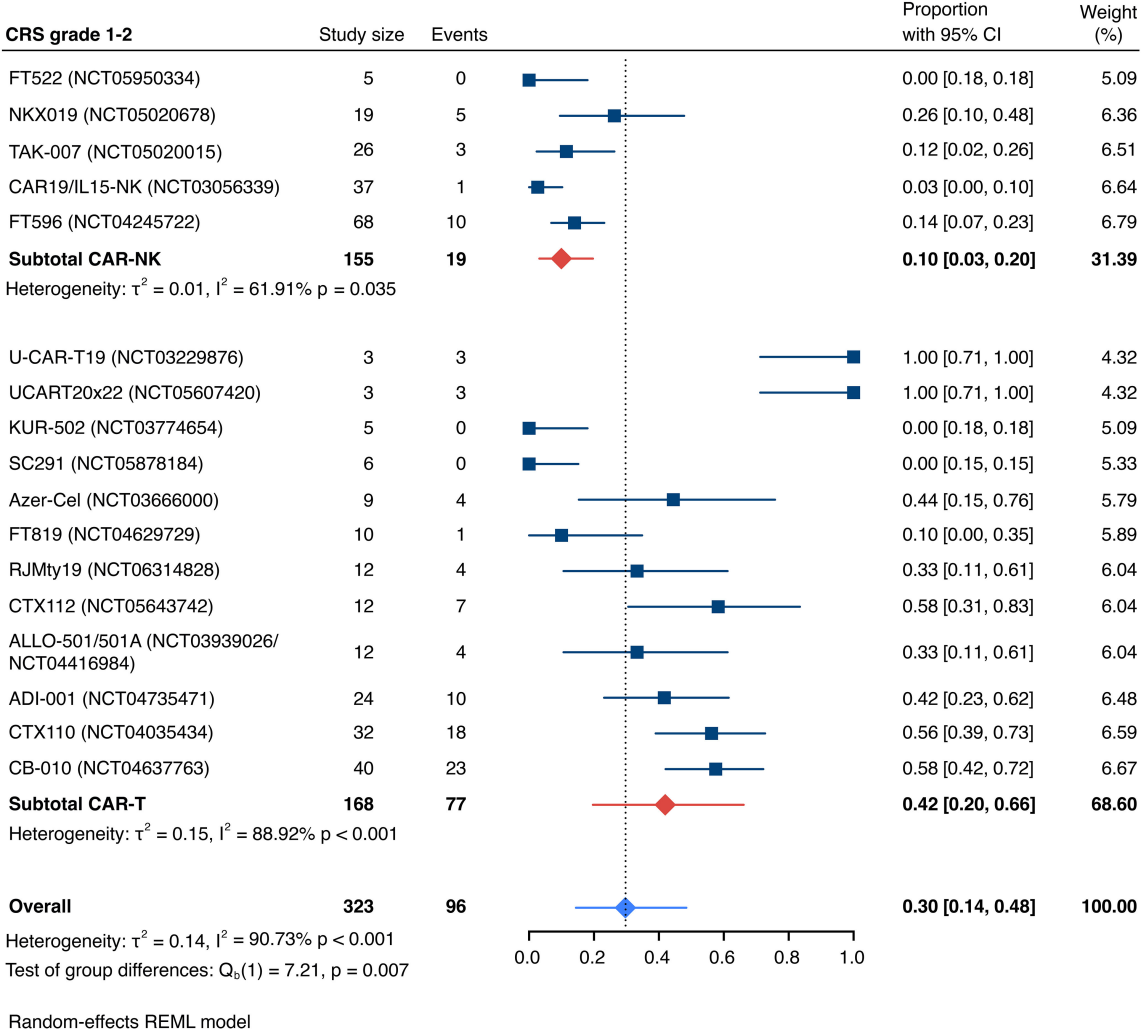
Forest plot illustrating the incidence of grade 1–2 cytokine release syndrome (CRS), stratified by cell source. Pooled estimates were computed for each subgroup and the overall weighted effect.

**Figure 6:**
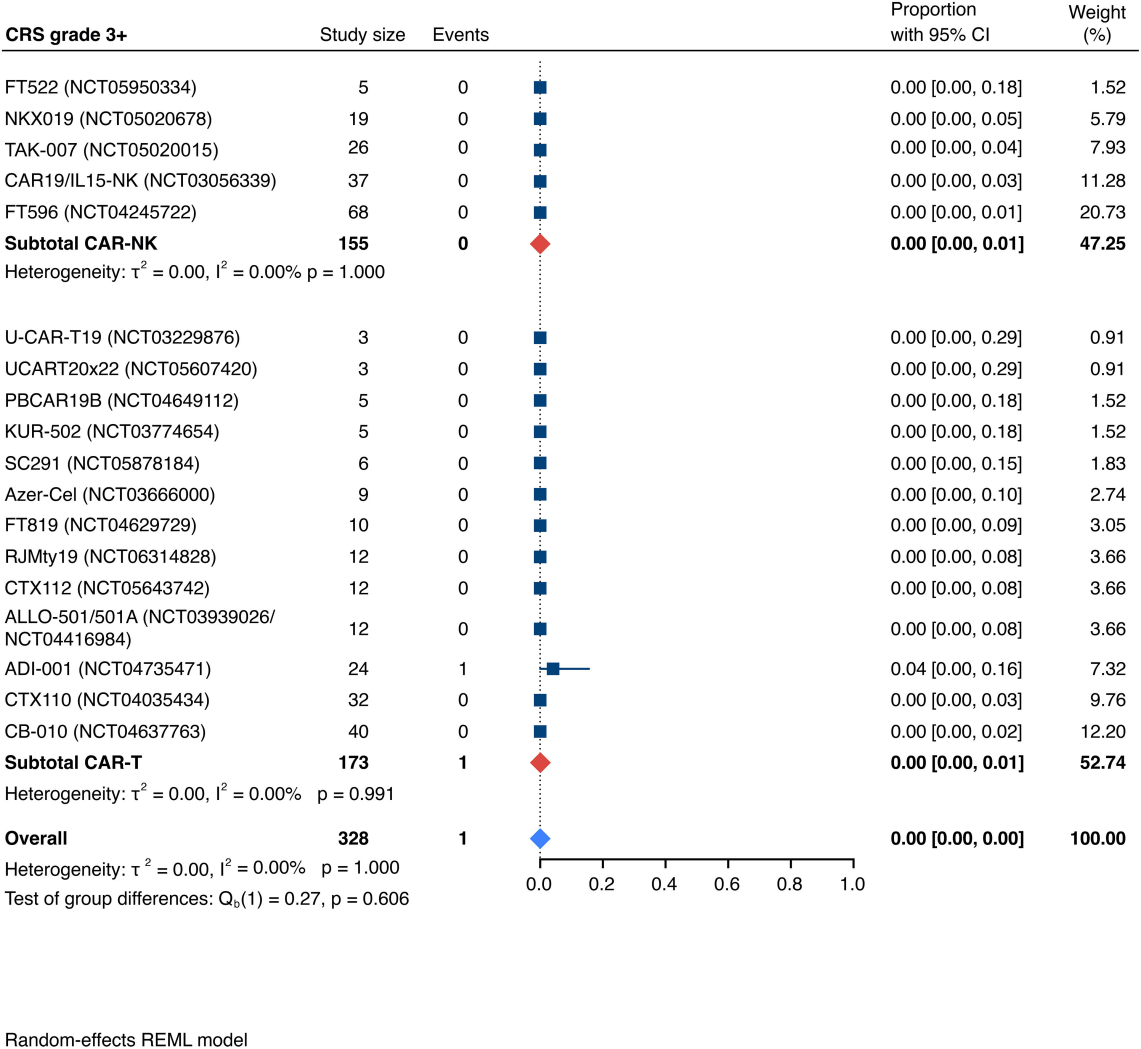
Forest plot illustrating the incidence of grade 3+ cytokine release syndrome (CRS), stratified by cell source. Pooled estimates were computed for each subgroup and the overall weighted effect.

**Figure 7:**
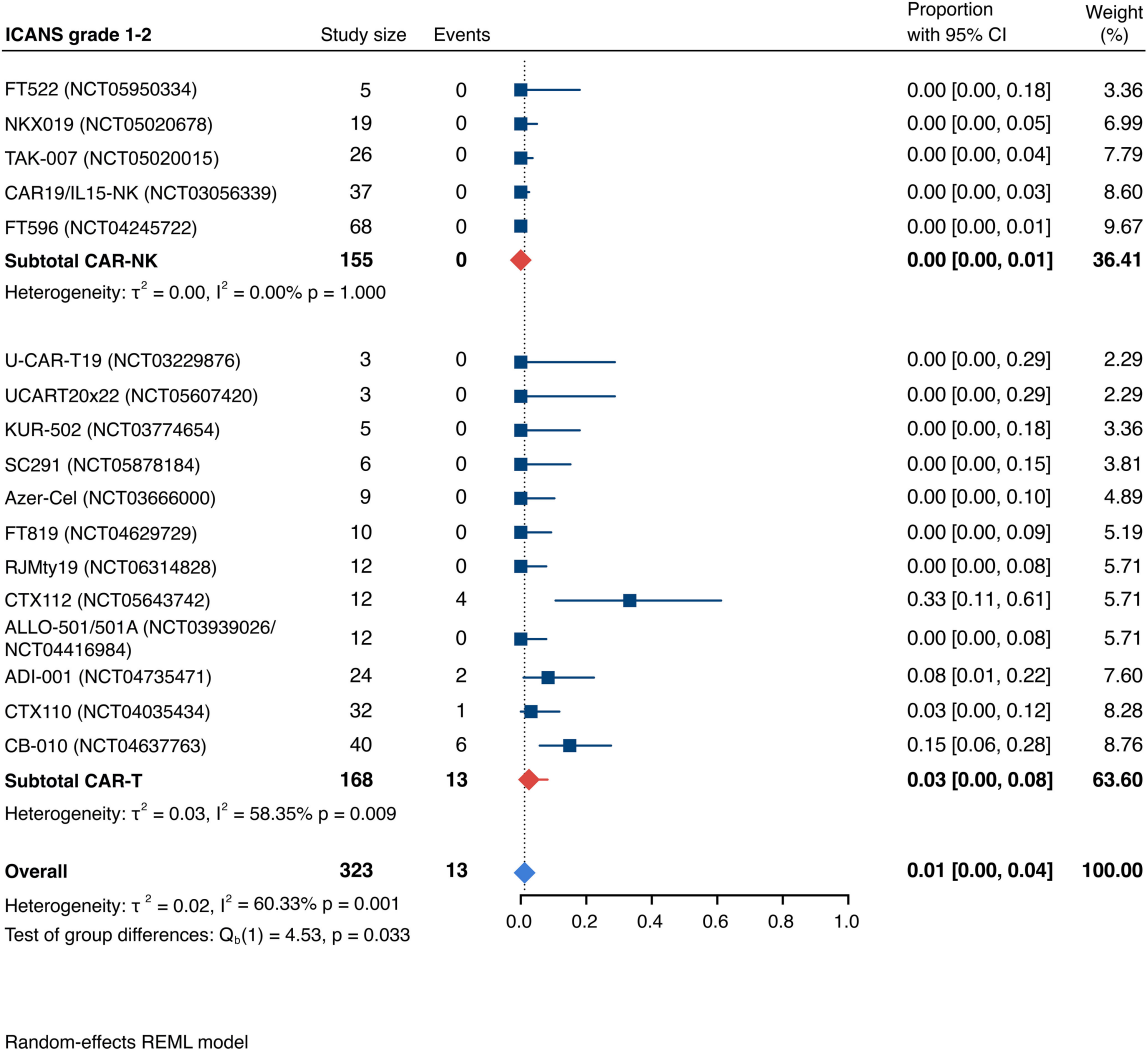
Forest plot illustrating the incidence of grade 1–2 immune effector cell-associated neurotoxicity syndrome (ICANS), stratified by cell source. Pooled estimates were computed for each subgroup and the overall weighted effect.

**Figure 8:**
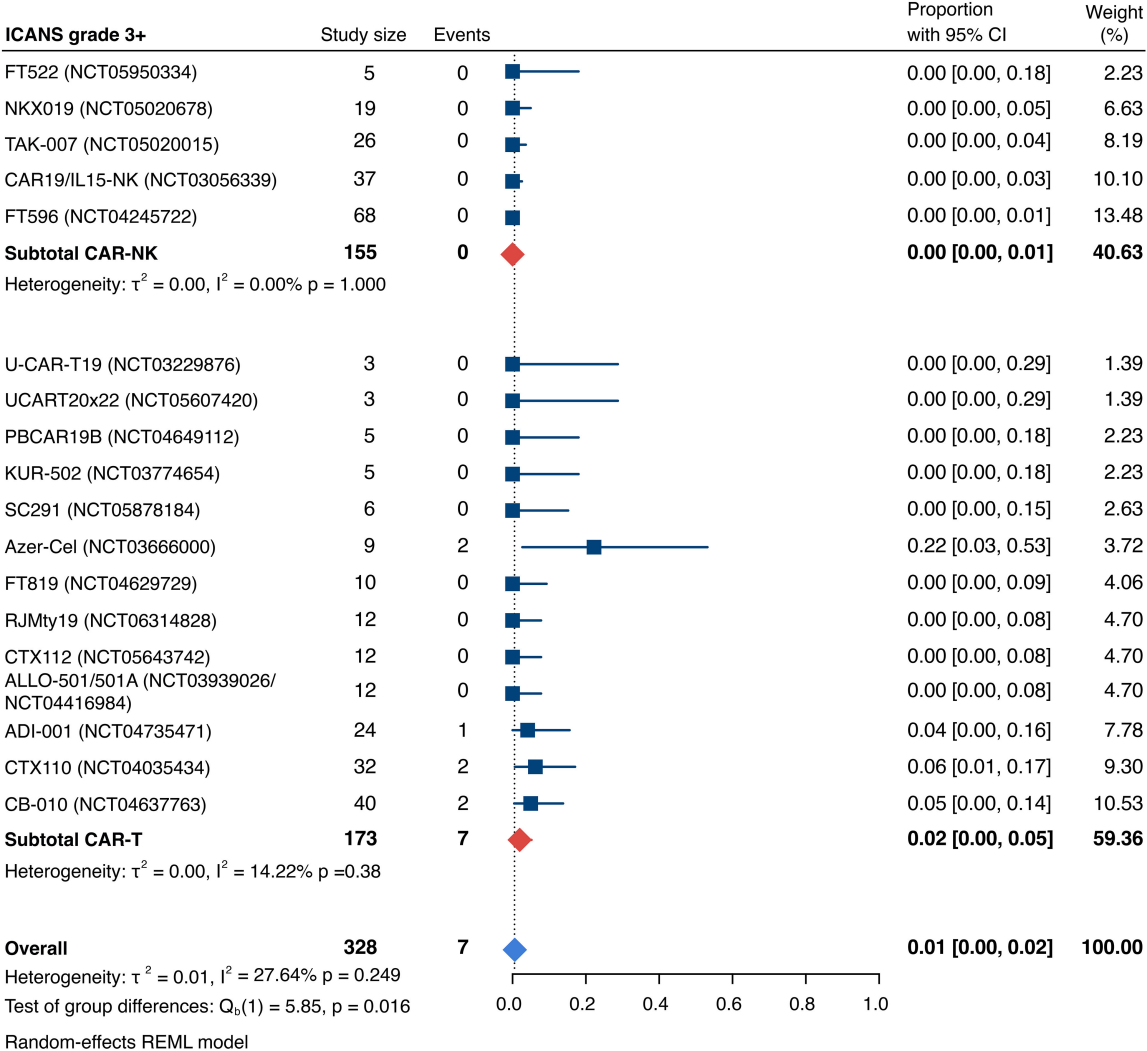
Forest plot illustrating the incidence of grade 3+ immune effector cell-associated neurotoxicity syndrome (ICANS), stratified by cell source. Pooled estimates were computed for each subgroup and the overall weighted effect.

Detailed severity grading for CRS and ICANS incidences was reported in all but two studies. The estimated overall incidence of low-grade CRS was 30% [95% CI, 14%-48%] (**Figure 5**), while severe CRS (grade 3+) occurred in 0.04% of patients [95% CI, 0.00%-0.49%] (**Figure 6**). The estimated overall incidence of low-grade ICANS was 1% [95% CI, 0%-4%] (**Figure 7**), with severe ICANS occurring in another 1% of patients [95% CI, 0%-2%] (**Figure 8**). No significant differences were observed in the incidence of high-grade CRS between patients infused with CAR-NK vs. CAR-T cell products (p=0.606). On the contrary, low-grade events of both CRS and ICANS as well as high-grade ICANS were significantly more frequent in CAR-T cell-infused patients (CRS grade 1-2: 42% [95%CI 20%-66%] vs. 10% [95%CI 3%-20%], n=323, p=0.007; ICANS grade 1-2: 3% [95%CI 0-8%] vs. 0% [95%CI 0-1%], n=323, p=0.033; ICANS grade 3+: 2% [95% CI 0%-5%] vs. 0% [95% CI 0%-1%], n=328, p=0.016).

**Figure 9:**
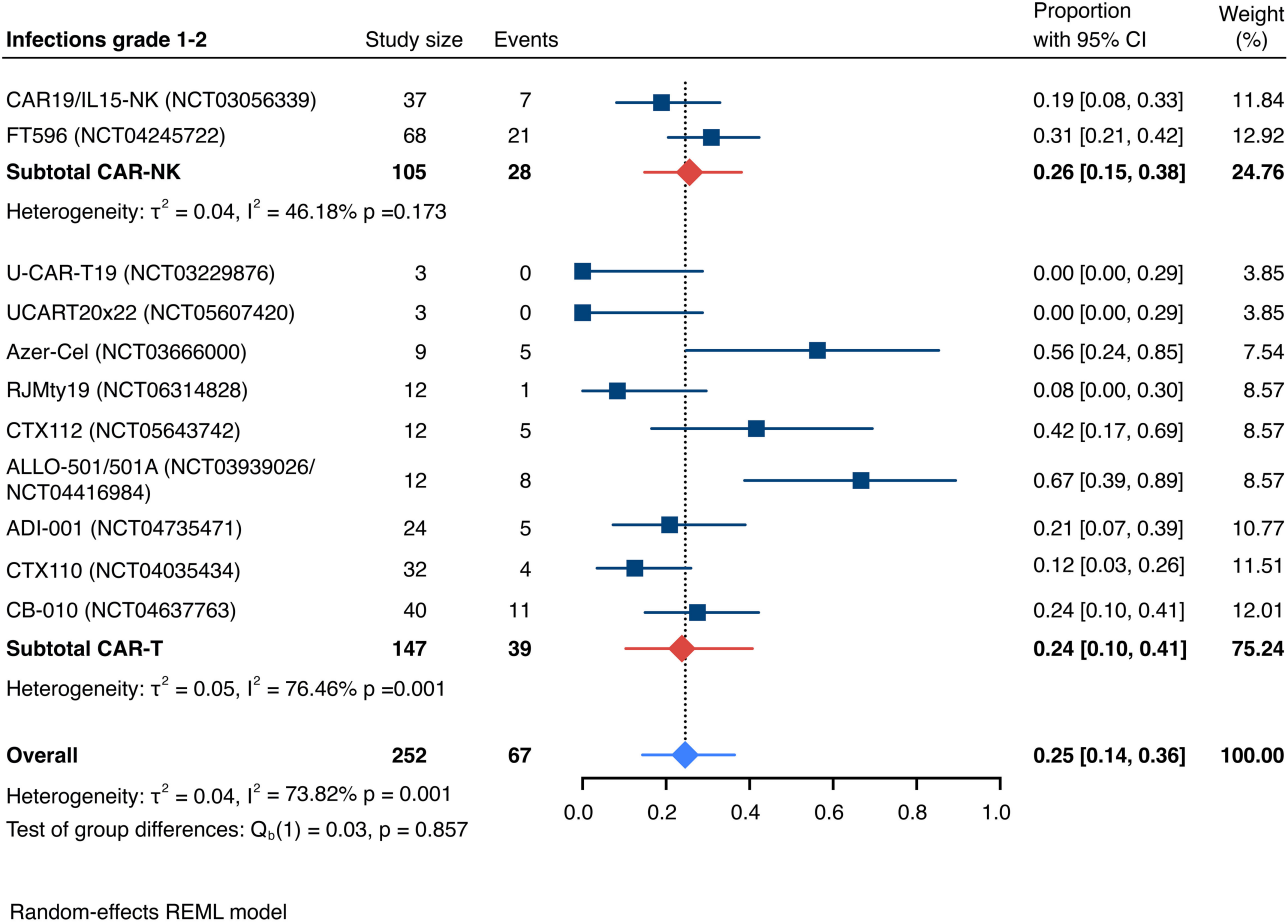
Forest plot illustrating the incidence of grade 1–2 infections, stratified by cell source. Pooled estimates were computed for each subgroup and the overall weighted effect.

**Figure 10:**
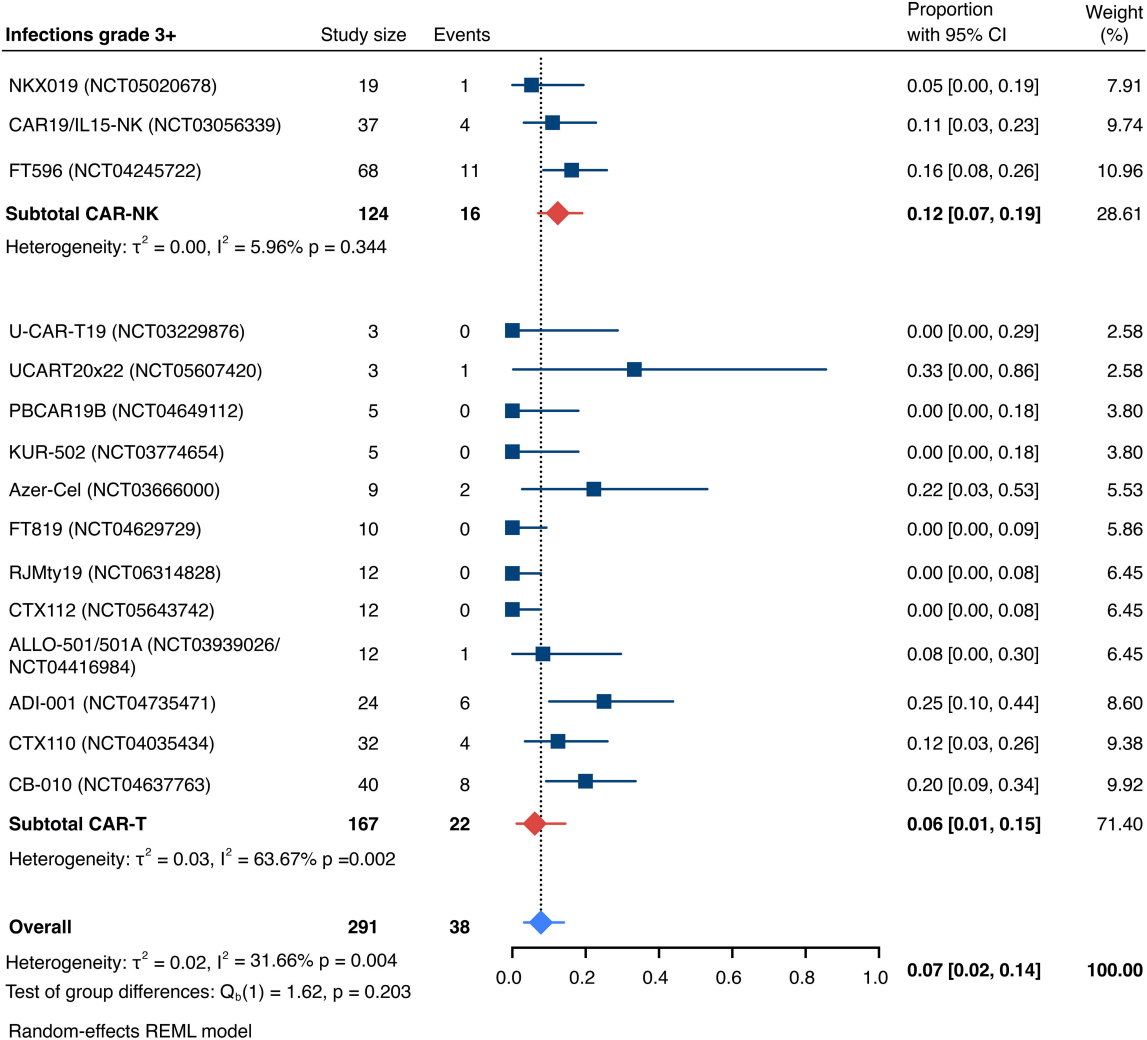
Forest plot illustrating the incidence of grade 3+ infections, stratified by cell source. Pooled estimates were computed for each subgroup and the overall weighted effect.

**Figure 11:**
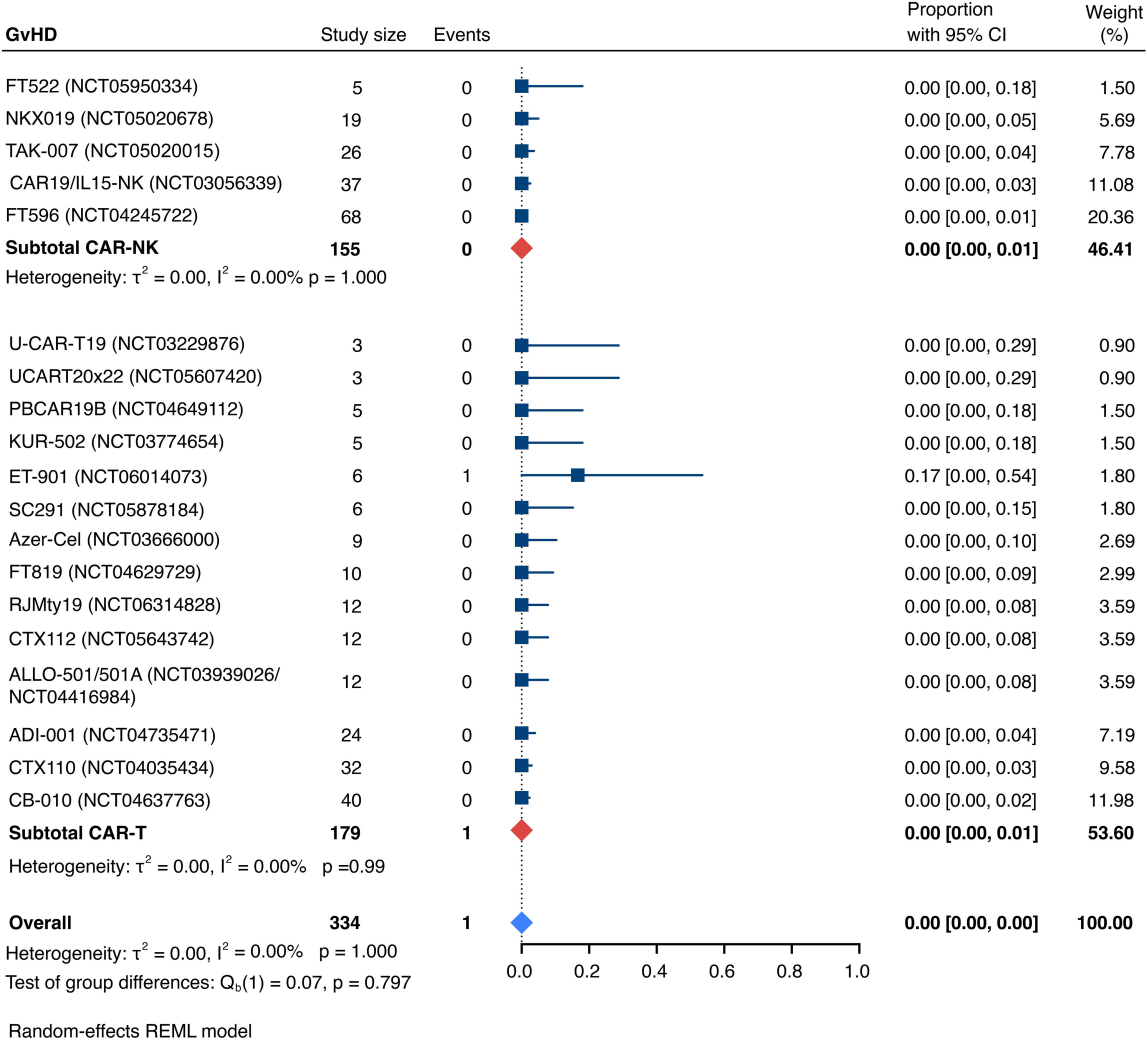
Forest plot illustrating the incidence of graft-versus-host disease (GvHD), stratified by cell source. Pooled estimates were computed for each subgroup and the overall weighted effect.

The incidence of low-grade and severe infections was 25% [95% CI, 14%-36%) (n=252) and 7% [95% CI, 2%-14%] (n=291), respectively, and no significant group-to-group differences were detected across investigated cell types (**Figures 9**, **10**). There was one case of a GvH-like reaction in a patient infused with ET-901 across a total of 334 infused patients, highlighting the impressive safety profile of CAR-engineered allogeneic cell therapies (**Figure 11**). Notably, there were no occurrences of GvHD in any of the administered HLA-mismatched CAR-NK cell products.

Dose-limiting toxicities (DLT) were reported for 3 patients including one death to HHV6 encephalitis post CTX110 infusion, one death possibly related to CB-010 infusion in the context of bladder perforation based on BK virus hemorrhagic cystitis and one DLN following PBCAR20A infusion not further classified by the trial sponsor.

### Emerging clinical trial landscape

Our search of clinical trial databases identified a total of 47 registered trials (24 recruiting trials; 8 trials active, not recruiting; 2 terminated trials; 3 withdrawn trials; 2 completed trials; 2 trials not yet recruiting; 6 trials with unknown status) investigating allogeneic CAR-modified immune effector cells for the treatment of relapsed/refractory hematologic malignancies, including r/r large B-cell lymphoma (LBCL). Of the identified trials, 32 are investigating investigational cell products targeting CD19, four trials are evaluating cell candidates directed at CD20, two candidates are targeting CD22, 2 candidates are targeting CD70, 3 investigational cell products are targeting CD30, and 4 trials are investigating multi-targeted cell products including CD19/CD20 (2), CD20/CD22 (1) and CD19/CD20/CD22 (1). The majority of trials (36/47) utilize T cells, which include cytotoxic T cells derived from PBMCs, T stem cell memory (Tscm), γδ T cells, double-negative T cells, and T cells derived from iPSCs. NK cells are investigated in 11 trials, derived from sources including PBMCs, CBMCs, the NK-92 cell line, and iPSCs. Overall, cell products are sourced from PBMCs (26), CBMCs (1 trial for NK cells), the NK-92 cell line (2 trials), iPSCs (1 trial each for T cells and NK cells), and EBV-specific T cells (4 trials). Trial sponsors were predominantly based in the US (22) and China (19) with four trials registered by a sponsor based in Switzerland, one based in France and another on in Australia. **Table 4** provides detailed information on the employed cell source, cellular engineering and gene editing strategies, sponsor and study status of identified trials.

**Table 4:** Clinical trial landscape of healthy third-party donor allogeneic CAR-engineered cellular therapeutics for relapsed and refractory B cell lymphoma (R/R B-NHL).

Product candidate	Trial	Sponsor	Disease	Cell source	Cellular engineering technology	Status
ThisCART19A	Phase 1, single-center, dose selection study *(*NCT05691153*)*	The First Affiliated Hospital of Soochow University	R/R B-NHL post autoCAR-T	T cells (PBMCs)	• CD19 CAR • Intracellular retention of TCR using a KDEL-tagged anti-CD3 single chain antibody (scFv) which prevents TCRαβ/CD3 from being secreted from the endoplasmic reticulum (ER) to prevent GvHD without gene editing	Recruiting, last update 2023-01-19
ThisCART19A	Phase 1, single-center, study (NCT05776407)	Chongqing University Cancer Hospital	R/R B-NHL	T cells (PBMCs)	• CD19 CAR • Intracellular retention of TCR using a KDEL-tagged anti-CD3 single chain antibody (scFv) which prevents TCRαβ/CD3 from being secreted from the endoplasmic reticulum (ER) to prevent GvHD without gene editing	Not yet recruiting, last update 2023-03-20
ThisCART19A	Phase 1 single arm, open-label, dose-escalation study (NCT05106907)	Fundamenta Therapeutics, Ltd.	R/R B-NHL	T cells (PBMCs)	• CD19 CAR • Intracellular retention of TCR using a KDEL-tagged anti-CD3 single chain antibody (scFv) which prevents TCRαβ/CD3 from being secreted from the endoplasmic reticulum (ER) to prevent GvHD without gene editing	Unknown status, last update 2021-11-04
ThisCART19A	Phase 1 signle arm trial (NCT05535673)	Zhengzhou University	R/R B-NHL	T cells (PBMCs)	• CD19 CAR • Intracellular retention of TCR using a KDEL-tagged anti-CD3 single chain antibody (scFv) which prevents TCRαβ/CD3 from being secreted from the endoplasmic reticulum (ER) to prevent GvHD without gene editing	Not yet recruiting, last update 2022-09-10
ThisCART22	*S*ingle arm, open-label, dose-escalation study (NCT05106946)	Fundamenta Therapeutics	R/R B-NHL	T cells (PBMCs)	• CD22 CAR • Intracellular retention of TCR using a KDEL-tagged anti-CD3 single chain antibody (scFv) which prevents TCRαβ/CD3 from being secreted from the endoplasmic reticulum (ER) to prevent GvHD without gene editing	Unknown status, last update 2021-11-11
PBCAR20A	Phase 1/2a, nonrandomized, open-label, parallel assignment, single-dose, dose-escalation, and dose-expansion study (NCT04030195)	Precision BioSciences	R/R B-NHL	T cells (PBMCs)	• CD20 CAR targeted to the *TRAC* locus, further details undisclosed	Completed, last update 2023-01-31
P-CD19CD20-ALLO1	Phase 1 study comprised of open-label, dose escalation and expansion cohort study (NCT06014762)	Poseida Therapeutics	R/R B-NHL	T stem cell memory (Tscm)	• Transposon-based DNA delivery system with large cargo capacity allows to target multiple antigens (CD19 & CD20) • TCR disruption to precent GvHD and partial B2M KO to mitigate host alloreactivity by Cas-CLOVER gene editing	Phase 1 data anticipated in 2025
CD19 CAR t-haNKs	Open-label phase 1 trial QUILT-3.092 (NCT05618925)	ImmunityBio	R/R B-NHL	NK-92 cell line cell line	• CD19 CAR • High-affinity variant of the human Fcγ receptor (FcγRIIIa/CD16a 158V) for enhanced ADCC • Endoplasmic reticulum-retained version of human interleukin-2 (ERIL-2) • Coadministration of N-803 NK/T cell activator and rituximab	Recruiting, last update 2024-08-20
CD19 CAR t-haNKs	Open-label phase 1 QUILT-106 trial (NCT06334991)	ImmunityBio	R/R B-NHL	NK-92 cell line cell line	• CD19 CAR • High-affinity variant of the human Fcγ receptor (FcγRIIIa/CD16a 158V) for enhanced ADCC • Endoplasmic reticulum-retained version of human interleukin-2 (ERIL-2) • Coadministration of N-803 NK/T cell activator and rituximab	Not yet recruiting, last update 2024-08-19
ATA3219	Phase 1 trial (NCT06256484)	Atara Bio	R/R B-NHL	EBV-specific T cells (CTLs) (PBMCs)	• CD20/CD19 scFv • 1XX costimulatory domain mitigates T cell exhaustion • EBV TCR	Initial clinical data anticipated Q1 2025
ATHENA-2 CAR T	Phase 1/2 trial (NCT06323525)	Chinese PLA General Hospital	R/R B-NHL	T cells (PBMCs)	• CD19 CAR • Preserved TCR (CD3 positive T cells) • Proprietary ‘Power3’ gene KO for enhanced persistence	Recruiting, last update 2024-12-02
Allogeneic CD19 CAR-T	Phase 1/2 trial (NCT05143112)	Shenzhen University General Hospital	R/R B-NHL	T cells (PBMCs)	• CD19 CAR • Further details undisclosed	Unknown status, last update 2021-12-03
Allogenic CD19-targeting CAR-γδT Cell	Phase 1/2 trial (NCT05554939)	Chinese PLA General Hospital	R/R B-NHL	γδT cells (PBMCs)	• CD19 (scFv FMC63).4-1BB.CD3ζ CAR • Further details undisclosed	Recruiting, last update 2024-11-29
Allogeneic CD30.CAR-EBVSTs	Phase 1 trial (NCT04288726)	Baylor College of Medicine	R/R B-NHL R/R HL R/R T-NHL R/R NK/T cell lymphoma	Healthy donor-derived Epstein Barr virus (EBV) specific T cells (EBVSTs)	• CD30.CD28.CD3ζ CAR	Recruiting, last update 2024-03-04
Allogeneic CD30.CAR-EBVSTs	Phase 1 trial (NCT04952584)	Baylor College of Medicine	R/R B-NHL R/R HL R/R T-NHL R/R NK/T cell lymphoma	Healthy donor-derived Epstein Barr virus (EBV) specific T cells (EBVSTs)	• CD30.CD28.CD3ζ CAR	Withdrawn, last update 2023-09-07
Constitutive IL7R (C7R) Modified Banked Allogeneic CD30.CAR EBVSTS	Phase 1 CABAL2 trial (NCT06176690)	Baylor College of Medicine	R/R B-NHL R/R HL R/R T-NHL R/R NKT cell lymphoma	Healthy donor-derived Epstein Barr virus (EBV) specific T cells (EBVSTs)	• CD30.CD28.CD3ζ CAR • Constitutively active IL7R (C7R) for deeper and longer anticancer efficacy • iCaspase9 safety switch	Not yet recruiting, last update 2024-10-26
ET-901 (ATHENA CAR T)	Phase 1/2 trial (NCT06014073)	Chinese PLA General Hospital	R/R B-NHL	T cells (PBMCs)	• CD19 (scFv FMC63).CD28. CD3ζ CAR • TRAC KO to prevent GvHD • Proprietary ‘GeneX’ KO for enhanced T cell persistence	Recruiting, last update 2023-09-11
KUR-502	Phase 1 ANCHOR2 trial (NCT05487651)	Athenex, Inc. (filed Chapter 11)	R/R B-NHL R/R ALL R/R CLL	NKT cells (PBMCs)	• CD19 CAR • IL-15 • shRNA to downregulate B2M and CD74	Recruiting, last update 2023-05-18
KUR-502	Phase 1 ANCHOR trial (NCT03774654)	Baylor College of Medicine	R/R B-NHL R/R ALL R/R CLL	NKT cells (PBMCs	• CD19 CAR • IL-15 • shRNA to downregulate B2M and CD74	Recruiting, last update 2024-09-19
LUCAR-20SP	Phase 1 trial (NCT06313957)	Peking University Cancer Hospital & Institute	R/R B-NHL	T cells (PBMCs)	• CD20 CAR • Further details undisclosed	Recruiting, last update 2024-11-15
LUCAR-20S	Phase 1 trial (NCT04176913)	The First Affiliated Hospital with Nanjing Medical University	R/R B-NHL	T cells (PBMCs)	• CD20 CAR • Further details undisclosed	Terminated, last update 2022-01-10 Results not published
SC262	Phase 1 VIVID trial (NCT06285422)	Sana Biotechnology	R/R B-NHL	T cells (PBMCs)	• CD22 CAR • TCR disruption • Disruption of MHC class I and II for host immune evasion • CD47 overexpression to evade innate immune recognition via Crispr-Cas12b editing	Recruiting, last update 2024-11-21
SC291	Phase 1 ARDENT trial (NCT05878184)	Sana Biotechnology	R/R B-NHL R/R CLL	T cells (PBMCs)	• CD19 CAR • TCR disruption • Disruption of MHC class I and II for host immune evasion • CD47 overexpression to evade innate immune recognition via Crispr-Cas12b editing	Active, not recruiting, last update 2024-11-12
CB-010	Phase 1 ANTLER study (NCT04637763)	Caribou Biosciences, Inc.	R/R B-NHL	T cells (PBMCs)	• CD19-CAR (scFv FMC63) targeted to *TRAC* locus replacing endogenous TCR to prevent GvHD • PD-1 KO to prevent premature CAR-T cell exhaustion and potentially enhance antitumor activity • CRISPR hybrid RNA-DNA (chRDNA) technology	Recruiting, last update 2024-11-21
ALLO-501	Phase 1 ALPHA trial (NCT03939026)	Allogene Therapeutics	R/R LBCL	T cells (PBMCs)	• CD19(scFv).4-1BB.CD3ζ CAR • TCR KO to prevent GvHD (utilizing TALEN gene editing) • CD52 KO to enable administration of anti-CD52 lymphodepleting antibodies (utilizing TALEN gene editing) • Rituximab recognition domain as kill switch	Active, not recruiting, last update 2023-12-01
Cema-cel (ALLO-501A)	Phase 1 ALPHA2 trial (NCT04416984)	Allogene Therapeutics	R/R LBCL	T cells (PBMCs)	• CD19(scFv).4-1BB.CD3ζ CAR • TCR KO to prevent GvHD (utilizing TALEN gene editing) • CD52 KO to enable administration of anti-CD52 lymphodepleting antibodies (utilizing TALEN gene editing)	Recruiting, last update 2024-06-06
CD19.AND.CD20. OR.CD22 CAR-T	Phase 1/2 trial (NCT03398967)	Chinese PLA General Hospital	R/R B-NHL R/R ALL R/R CLL	T cells (PBMCs)	• CD19 AND CD20 OR CD22 CAR • Further details undisclosed	Unknown status, last update 2018-01-16
CTA30X UCAR-T cells	Phase 1 trial (NCT05015972)	920th Hospital of Joint Logistics Support Force of People’s Liberation Army of China	R/R B-NHL	T cells (PBMCs)	• CD19 CAR • Further details undisclosed	Withdrawn, last update 2022-11-19
Azer-cel (PBCAR0191)	Phase 1/2a, nonrandomized, open-label, parallel assignment, dose-escalation, and dose-expansion study (NCT03666000)	Imugene Limited	R/R B-NHL	T cells (PBMCs)	• CD19 CAR targeted to the *TRAC* locus utilizing ARCUS gene editing	Recruiting, last update 2024-12-16
UCART019	Phase ½ trial (NCT03166878)	Chinese PLA General Hospital	R/R B-NHL R/R ALL R/R CLL	T cells (PBMCs)	• CD19 CAR • Further details undisclosed	Unknown status, last update 2017-06-23
UCART20x22	Phase 1/2a NatHaLi-01 clinical trial (NCT05607420)	Cellectis	R/R B-NHL	T cells (PBMCs)	• CD20 CAR + CD22 CAR • TRAC KO to prevent GvHD • CD52 KO to enable use of anti-CD52 mAbs • TALEN gene editing	Recruiting, last update 2024-03-10
CTX130	Phase 1 COBALT-LYM trial (NCT04502446)	CRISPR Therapeutics AG	R/R B- and T cell malignancies	T cells (PBMCs)	• CD70 CAR targeted to the *TRAC* locus • B2M KO	Active, not recruiting, last update 2024-05-21 Clinical results published (n=41 pts with T cell malignancies)
CTX112	Open-label, multicenter, Phase 1/2 study (NCT05643742)	CRISPR Therapeutics	R/R B-NHL	T cells (PBMCs)	• CD19 CAR KI into *TRAC* locus using an AAV template • TCR disruption to prevent GvHD • B2M KO to eliminate MHC1 expression • TGFBR2 KO to overcome immunosuppression in the TME • YC3H12A (Regnase-1) to increase cell expansion and functional persistence	Recruiting, last update 2024-08-28
CTX110	Phase 1 CARBON open-label, multicenter, study (NCT04035434)	CRISPR Therapeutics	R/R B-NHL	T cells (PBMCs)	• CD19 CAR • TCR disruption to prevent GvHD • B2M KO to eliminate MHC1 expression	Active, not recruiting, last update 2024-03-21
CTX131	Phase ½ trial (NCT06492304)	CRISPR Therapeutics	R/R hematologic malignancies	T cells (PBMCs)	• CD70 CAR KI into *TRAC* locus using an AAV template • TCR disruption to prevent GvHD • B2M KO to eliminate MHC1 expression • TGFBR2 KO to overcome immunosuppression in the TME • CD70 KO to prevent fratricide • YC3H12A (Regnase-1) to increase cell expansion and functional persistence	Recruiting, last update 2024-11-29
PACE CART19	Phase 1 trial (NCT05037669)	University of Pennsylvania	R/R B-NHL R/R ALL R/R CLL	T cells (PBMCs)	• CD19 CAR • TCR disruption • HLA class I and II disruption	Withdrawn, last update 2023-06-22
(n)U-CAR-T19	Phase 1 clinical trial (NCT03229876)	Bioray Laboratories	R/R B/ALL and R/R B-NHL	T cells (PBMCs)	U-CAR-T19: • CD19(scFv FMC63).4-1BB.CD3ζ CAR • TRAC KO to prevent GvHD B2M KO to disrupt MHC1 and prevent rejection by alloreactive T cells • CD19(scFv FMC63).4-1BB.CD3ζ CAR • TRAC KO to prevent GvHD • HLA-A/B KO to resist NK cell alloreactivity	Suspended, last update 2023-07-24, clinical results reported
CD19 CAR NK	Phase 1 single arm, open-label trial (NCT05673447)	Shanghai Hospital	R/R DLBCL	NK cells (undisclosed source)	• CD19 CAR, further details undisclosed	Recruiting, last update 2023-08-28
CD19-CAR-NK	Phase 1 open label, single arm trial (NCT05739227)	Xuzhou Medical University	R/R B-NHL R/R ALL R/R CLL	NK cells (undisclosed source)	• CD19 CAR, further details undisclosed	Recruiting, last update 2023-02-22
NKX019-101	Open label, multicenter Phase 1 trial (NCT05020678)	Nkarta	R/R B-NHL	NK cells (PBMCs)	• Humanized CD19(FMC63 scFv).OX40.CD3ζ CAR • Membrane-bound IL-15 for autocrine growth support and enhanced persistence	Recruiting, last update 2024-03-21
TAK-007	Phase 2 trial (NCT05020015)	Takeda	R/R B-NHL	NK cells (CBMCs)	• CD19.CD28. CD3ζ CAR • Inducible Caspase9 safety switch • IL-15 cytokine support	Active, not recruiting, last update 2024-05-29
PCAR-119	Phase 1/2 trial (NCT02892695)	PersonGen BioTherapeutics (Suzhou) Co., Ltd.	CD19+ R/R hematologic malignancies	NK cells (undisclosed source)	• CD19 CAR, further details undisclosed	Unknown status, last update 2016-12-06
QN-019a	Phase 1 trial (NCT05379647)	Zhejiang University	R/R B-NHL R/R ALL	NK cells (undisclosed source)	• CD19 CAR, further details undisclosed	Unknown status, last update 2022-05-18
FT522	Phase 1 multicenter trial (NCT05950334)	Fate Therapeutics	R/R B-NHL	NK cells (iPSCs)	• CD19-CAR CD3ζ • High affinity, non-cleavable CD16 Fc receptor (hnCD16) • CD38 KO to prevent NK cell fratricide, enhance metabolic fitness • IL-15/IL-15 receptor fusion • Alloimmune-defense receptor (ADR) targeting 4-1BB	Recruiting, last update 2024-07-26
FT819	Phase I dose-finding study (NCT04629729)	Fate Therapeutics	R/R B-NHL	T cells (iPSCs)	• 1XX CAR19 with CD28 costimulatory domain and modified CD3ζ targeted to the *TRAC* locus • Biallelic disruption of *TRAC* at clonal level to delete endogenous TCR to prevent GvHD • CD58 KO • Alloimmune-defense receptor (ADR) targeting 4-1BB	Active, not recruiting, last update 2024-08-02
RJMty19	Open-label, single-dose, phase 1 study (NCT05453669)	Guangdong Ruishun Biotech Co., Ltd	R/R high-grade B-NHL	Double-negative T cells (PBMCs)	• Humanized CD19(scFv).4-1BB.CD3ζ CAR	Recruiting, last update 2022-07-12
ADI-001	Open-label, multi-center phase 1 GLEAN trial (NCT04735471)	Adicet Bio	High grade R/R B-NHL	γΔ T cells (PBMCs)	• CD20 CAR • No gene editing	Active, not recruiting, last update 2024-09-19

ADCC, antibody-dependent cellular cytotoxicity; Azer-cel, Azercabtagene zapreleucel; Cema-cel, Cemacabtagene ansegedleucel; mAb, monoclonal antibody.

### Risk of bias

Most of studies the studies included in this systematic review and meta-analysis are early-phase open-label trials, including single center studies which lacked estimates of random variability and blinded outcome assessors. This limitation underscores the need for more robust, multicenter, randomized, and blinded studies to validate these early findings.

## Discussion

We conducted, for the first time, a systematic review and meta-analysis to evaluate the emerging clinical evidence on the safety and antitumor efficacy of allogeneic CAR-engineered cell products for the treatment of large B-cell lymphoma (LBCL). Nineteen studies met the inclusion and exclusion criteria, encompassing a total of 334 patients evaluable for safety (155 receiving CAR-NK therapy and 179 receiving CAR-T therapy) and 235 patients evaluable for response (77 receiving CAR-NK therapy and 158 receiving CAR-T therapy). The pooled estimates for the best overall response rate (bORR) and the best complete response rate (bCRR) were 52.5% [95% CI, 41.0-63.9] and 32.8% [95% CI, 24.2-42.0], respectively. This study highlights the exceptional safety profile of allogeneic cell therapies. Across all included studies, only one case of grade 3+ cytokine release syndrome (CRS) was reported, the incidence of grade 3+ immune effector cell-associated neurotoxicity syndrome (ICANS) was 1% [95% CI, 0%-2%], and one occurrence graft-versus-host like reaction (1/334) was reported. Intriguingly, the investigated CAR-NK cell candidates exhibited an even more favorable safety profile compared to allogeneic CAR-T cells with a lower incidence of grade 1–2 CRS (10% [95% CI, 3-20] vs. 42% [95% CI, 20-66]), further underscoring the tolerability of NK cells in the allogeneic setting. Unlike T cells, NK cells naturally tolerate HLA mismatches and have demonstrated excellent safety across numerous clinical studies using unmatched allogeneic NK cell products (63–72). These favorable immunobiological properties are likely due to their germline-encoded repertoire of activating and inhibitory receptors, which finely balance responsiveness and restraint, integrating diverse signals to mediate targeted cytotoxicity without provoking systemic immune overactivation or uncontrolled cytokine release.

Together, these findings highlight the therapeutic potential of allogeneic cell products in r/r LBCL with a significantly reduced toxicity burden compared to their autologous contenders, making them a highly attractive and cost-effective treatment option for this difficult-to-treat patient population, including in the outpatient setting.

### Strengths and limitations

The presented study has several notable strengths. First, it is the first study to systematically investigate and compare multiple competing allogeneic cell products aiming to upend the standard-of-care for a patient population with a high unmet medical need. Second, this study incorporates the most current data, including findings presented at recent medical conferences, making it a valuable and up-to-date resource that provides a critical perspective on the emerging clinical trial landscape of allogeneic cell therapies. Third, it aims to harmonize the analyzed patient groups as much as possible despite variability in the underlying patient populations. By stratifying analyses based on cell source, this study provides a meaningful comparison between CAR-NK and CAR-T cell therapies, ensuring nuanced insights into their respective efficacy and safety profiles.

Nevertheless, several limitations must be acknowledged. These are largely inherent in the analyzed primary studies, which often included small cohorts and were conducted in early-phase clinical trial settings. Many of these studies were dose-escalation and dose-expansion trials, where response rates observed during the dose-escalation phase may understate the true effect size due to suboptimal dosing. What is more, limited follow-up times of included trials and variable reporting standards restrict the assessment of therapeutic durability. Additionally, the limited number of studies available and the relatively small overall sample size restrict the generalizability of the findings. Variability in the underlying patient populations further complicates pooled analyses. Differences include the number of prior therapies received (e.g., prior exposure to CD19-targeted therapeutics, prior CAR-T exposure, and prior allogeneic hematopoietic stem cell transplantation), international Prognostic Index (IPI) risk scores, and age. This heterogeneity warrants cautious interpretation of pooled estimates of response and safety particularly across investigated groups.

Lastly, the presented allogeneic CAR-T and CAR-NK cell candidates differ with respect to their respective CAR design including costimulatory domains, employed gene editing platforms and inclusion of additional genetic edits and molecular payloads.

### Allogeneic vs. autologous CD19-directed CAR-T: real-world evidence

Recent real-world studies have demonstrated overall and complete response rates in the range of 58-84% and 39-68%, respectively, in patients with r/r LBCL receiving commercially available autologous CAR-T cell products (8, 9, 15, 18, 19). However, these responses rates come at the expense of significant toxicities, with CRS and ICANS occurring in 52-88% and 30-56% of cases, respectively. Higher-grade toxicities (grade ≥3) were observed in 3-23% of cases for CRS and 6-23% for ICANS, underscoring the potential for severe and life-threatening adverse effects. In contrast, our meta-analysis of allogeneic CAR-IEC products showed markedly lower rates of low-grade (grade 1-2) CRS (30%) and ICANS (1%). High-grade CRS and ICANS were rare, with only 1 and 7 cases, respectively, among 328 infused patients. In terms of response, early-phase clinical trial readouts demonstrated encouraging efficacy signals for allogeneic CAR-IEC candidates with pooled estimates for best ORR and best CRR of 52.5% [95% CI, 41.0-63.9] and 32.8% [95% CI, 24.2-42.0], respectively. Beyond their favorable safety profile and promising efficacy, allogeneic CAR-T and CAR-NK cell therapies have the potential to significantly reduce treatment costs and improve accessibility compared to their autologous counterparts.

In principle, gene-editing strategies–while essential for enabling allogeneic use of T cells–may inadvertently compromise cellular efficacy and impinge on clinical response rates. In particular, the requirement for TRAC knockout in alloCAR-T cells, designed to prevent GvHD, eliminates endogenous TCR signaling, which plays a critical role in activation-induced clonal expansion and persistence. Loss of this native signaling axis may reduce the durability and proliferative capacity of the infused product, especially in the absence of supportive cytokine cues.

However, direct comparisons of response rates remain challenging due to fundamental differences in treatment paradigms and differences in examined patient cohorts. Allogeneic cell products eliminate the need for leukapheresis, a process that may fail to yield high-quality starting material in patients with low lymphocyte counts or dysfunctional lymphocytes with impaired fitness due to prior chemoimmunotherapy, tumor-induced immunosuppression, or inherent T cell defects, such as those seen in HIV-associated DLBCL (73).

This distinction makes direct comparison difficult, as most autologous CAR-T cell studies report outcomes only for patients who successfully received the cells (modified intention-to-treat, mITT) rather than from the initial screening population (intention-to-treat, ITT). Furthermore, the time required for CAR-T cell manufacturing often favors patients with less aggressive disease, as those able to wait for production may inherently have a better prognosis (74). Conversely, the “off-the-shelf” nature of allogeneic therapies allows for more rapid treatment initiation, which may better address patients with rapidly progressing disease.

One study directly compared response rates between CAR-NK cells and autologous CAR-T therapies on an ITT basis. When analyzed on an ITT basis, the reported complete response (CR) rate for autologous CAR-T in DLBCL was 34% (95% CI: 27–42%), which was similar to the 27.8% (95% CI: 10–53%) CR rate observed with CAR-NK cells in the same study (31).

This comparison underscores the importance of considering ITT analyses when evaluating the relative efficacy of allogeneic and autologous therapies. Moreover, a number of included studies enrolled patients who relapsed after autologous CD19 CAR-T cell therapies, further complicating any direct comparisons due to inherent differences in the examined patient populations. As allogeneic CAR-engineered cell products, including CAR-NK cells, advance through clinical development and potentially move into earlier lines of therapy, it will be particularly interesting to monitor how their response rates evolve in broader and less heavily pretreated patient populations. Early-line settings may reveal additional benefits of allogeneic products, such as shorter manufacturing times, elimination of leukapheresis, and broader applicability due to reduced cost.

### Ongoing trials and expanding targets

In addition to the studies included in this analysis, several clinical trials are currently investigating allogeneic CAR-T and CAR-NK cell therapies in patients with r/r LBCL. These ongoing trials aim to further evaluate the safety, efficacy, and applicability of these therapies across diverse patient populations. **Table 4** provides an overview of these ongoing trials, highlighting their design, target populations, and therapeutic approaches.

While the majority of investigational cell products target CD19, other targets for allogeneic CAR therapies are actively being explored. Notably, recent studies of allogeneic CAR-T cells targeting CD70 have demonstrated promising safety and efficacy signals in renal cell carcinoma (RCC) (75, 76) and T-cell non-Hodgkin lymphoma (T-NHL) (77). These findings underscore the expanding potential of allogeneic CAR-based therapies across both hematologic malignancies and solid tumors, paving the way for broader applications of these innovative treatments.

### Cell sources and donor selection

The choice of cell sources and donor selection are pivotal in the development of allogeneic CAR-T and CAR-NK cell therapies, influencing scalability, functionality, and clinical outcomes. Peripheral blood mononuclear cells (PBMCs) from healthy donors remain a widely utilized source for allogeneic CAR-T and CAR-NK therapies. These cells offer robust cytotoxic activity and the ability to generate multiple doses from a single donor, enabling rapid treatment delivery and cost-effectiveness. However, reliance on healthy donors introduces variability in cell quality and functionality, prompting exploration of alternative sources.

Cord blood has emerged as a promising source for CAR-NK cell manufacturing and various strategies of IL-15 cytokine armoring enable long-term *in vivo* persistence of engineered NK cells. Cord blood–derived NK cells demonstrate potent innate cytotoxicity and lack the propensity to induce graft-versus-host disease (GvHD). Recent research has further optimized the use of cord blood by identifying factors that predict enhanced NK cell functionality. A study found that nucleated red blood cell (NRBC) count and time from collection to cryopreservation are key determinants of cord blood unit (CBU) quality. Specifically, CBUs with an NRBC count ≤8×10^7^ and a collection-to-cryopreservation time ≤24 hours correlated with superior NK cell cytotoxicity. These findings support rational donor selection and processing strategies to maximize the therapeutic potential of cord blood–derived NK cells, making them an increasingly attractive option for clinical application (31).

Cell-line–derived platforms, such as high-affinity NK (haNK) cells, have also been explored as alternatives to donor-dependent sources. HaNK cells are engineered with high-affinity CD16 (FcγRIIIa) to enhance antibody-dependent cellular cytotoxicity (ADCC) and are equipped with IL-2 armoring to improve persistence. Due to their origin, haNK cells require irradiation prior to infusion, potentially restricting *in vivo* persistence (42). Currently, CD19-targeted high affinity NK cells (t-haNKs) are being clinically evaluated in two phase 1 clinical trials (NCT05618925 and QUILT 106).

Induced pluripotent stem cell (iPSC) technology offers another innovative approach to cell sourcing. iPSC-derived NK cells are created from clonal master-engineered iPSC lines. These cells can be produced in large, standardized quantities with consistent quality, enabling off-the-shelf availability and addressing batch variability (30, 39–41). Beyond NK cells, iPSC-derived T cells, such as FT819, are being developed as potential homogeneous CAR-T cell products for clinical use (58, 78). By leveraging genetic engineering, iPSC platforms provide a renewable and versatile option for producing highly customized cell therapies, though their broader clinical applicability remains under investigation.

Lastly, Epstein-Barr virus (EBV)-specific cytotoxic T lymphocytes (CTLs) have been investigated as an allogeneic cell platform for CAR T-cell therapeutics due to their established safety profile positioning them as a potentially versatile and immunocompatible cellular backbone for genetically engineered CAR T-cell therapies.

The diversification of cell sources underscores the growing flexibility and innovation in allogeneic cell therapy development. From PBMCs and cord blood to iPSC-derived platforms and cell lines, each source offers unique advantages and challenges. Of note, the use of healthy donor-derived allogeneic cell sources may provide a critical safety benefit as healthy donors presumably are less likely to harbor clonal hematopoiesis or predisposing genetic lesions and have not undergone prior exposure to genotoxic antineoplastic agents. Going forward, rational donor selection could exclude donors with clonal hematopoiesis, thereby reducing the theoretical risk of therapy-related secondary malignancies as have been observed in a limited number of autologous CAR-T products. As the field advances, the optimization of cell sources and donor selection will play a crucial role in improving accessibility, functionality, and therapeutic outcomes for patients with hematologic malignancies and beyond.

### Emerging immune cell types for CAR engineering

In addition to CAR-T, CAR-NKT and CAR-NK therapies, CAR-macrophages are emerging as another exciting avenue in cellular immunotherapy (79–84). Macrophages, as innate immune cells with a natural ability to infiltrate solid tumor microenvironments, represent a unique therapeutic platform. Unlike T or NK cells, macrophages can remodel the tumor microenvironment, phagocytose cancer cells, and stimulate secondary immune responses through antigen presentation. CAR-macrophages are engineered to enhance their ability to specifically target tumor cells and sustain immune activation within the immunosuppressive tumor milieu.

Although CAR-macrophage therapies are still in preclinical or early-phase clinical development, their potential to overcome some of the limitations associated with other cell types has garnered significant attention. Preclinical studies have shown promising antitumor activity in both hematologic and solid tumor models. The first clinical data for HER2-directed CAR-macrophages displayed encouraging safety data in patients with recurrent and metastatic solid turmors (81), promising to expand the arsenal of CAR-based treatments and potentially address indications where other immune cell types have shown limited efficacy.

### Gene editing platforms and cellular engineering strategies

To enable the safe clinical application of allogeneic CAR-T cell therapies, overcoming graft-versus-host disease (GvHD) and host-versus-graft (HvG) responses remains a central challenge requiring precise gene-editing strategies. To prevent GvHD, most allogeneic CAR-T designs rely on targeted disruption of the *TRAC* locus, eliminating TCR expression thereby abrogating GvHD risk.

Alloreactive rejection (HvG) of infused CAR-T cell products, driven primarily by αβ T cells targeting mismatched class I molecules, may be circumvented by knocking out β2-microglobulin (B2M), a crucial subunit of HLA class I, thereby, shielding donor cells from cytotoxic CD8+ T cells. However, this approach has the potential to trigger NK cell-mediated CAR-T lysis which in turn has been addressed by HLA-E overexpression, thereby inhibiting NK cell activation via the NKG2A receptor. Another strategy to mitigate NK cell-mediated CAR-T cell rejection is by HLA-A/B deletion. Additional modifications include CD47 overexpression to inhibit macrophage-mediated phagocytosis and CD52 knockout to protect donor cells from depletion by alemtuzumab-based lymphodepletion regimens.

Besides circumventing alloreactivity, cell engineering strategies are similarly being employed to prevent fratricide, for instance in CD70-targeting CAR-T cells (85) or to augment cellular fitness by targeted deletion of functional checkpoints, including PD-1 (46) and other previously unrecognized negative immune regulators which have recently emerged from large-scale CRISPR screening efforts in primary immune cells (86–90).

Lastly, safety switches in form of rituximab recognition domains (29) or inducible Caspase9 (21, 22, 31) are incorporated into some cell products to enable rapid *in vivo* elimination of engineered cells the event of inadvertent toxicities.

Multiple gene-editing platforms enable these modifications, each with unique strengths and limitations. Zinc finger nucleases (ZFNs) and transcription activator-like effector nucleases (TALENs) are early-generation technologies that induce double-stranded breaks (DSBs) at specific loci. ZFNs rely on protein-DNA interactions but face challenges related to off-target effects and complex design. TALENs offer greater specificity but require labor-intensive customization.

The CRISPR-Cas system revolutionized gene editing by introducing RNA-guided targeting, enabling efficient multiplex editing. However, concerns persist about off-target effects, chromosomal rearrangements, and translocations following DSB-induced repair. To reduce these risks, base editing has emerged, allowing precise single-nucleotide changes without inducing DSBs. This approach enhances safety while maintaining editing efficiency.

Proprietary ARCUS nucleases, based on engineered homing endonucleases, offer an alternative platform with a single-cut mechanism and enhanced specificity. ARCUS’ small size enables easier delivery into cells, while its reduced off-target profile promises improved genomic stability. As these platforms continue to evolve, balancing precision, scalability, and clinical feasibility will be essential for advancing next-generation allogeneic adoptive cell therapies.

### Positioning against bispecific antibodies

Allogeneic CAR-equipped cell products must also strategically position themselves against a growing number of bispecific antibodies, which are rapidly becoming an important therapeutic option in r/r LBCL. Bispecific antibodies, such as epcoritamab (CD3×CD20) (91, 92) and glofitamab (CD3×CD20) (93–95), have shown promise in providing effective, off-the-shelf treatments with lower manufacturing complexity and reduced treatment costs. These agents also tend to be associated with fewer severe adverse events, such as CRS or ICANS, further enhancing their appeal as practical alternatives (91–95).

However, bispecific antibodies may come with a trade-off in terms of long-term efficacy, particularly progression-free survival (PFS). Recent studies suggest that while bispecifics achieve impressive initial response rates, their durability may not match that of CAR-T therapies, which benefit from the ability of engineered cells to persist and provide sustained immune surveillance (37, 38). As allogeneic CAR-T and CAR-NK therapies continue to evolve, demonstrating superior durability of responses and the ability to achieve long-term remissions will be critical for distinguishing themselves in an increasingly competitive treatment landscape.

### Impact of therapeutic sequencing

The sequencing of therapeutic agents, particularly bispecific antibodies, autologous CAR-T cells, and allogeneic CAR-T cells, plays a critical role in determining response rates and optimizing outcomes for patients with r/r LBCL. Exposure to previous CD19-targeting agents, including tafasitamab (96), a CD19-targeting monoclonal antibody, and loncastuximab tesirine (97), a CD19-directed antibody-drug conjugate, may exert selective pressure that promotes antigen escape, potentially complicating treatment in later-line patients and warranting the exploration of additional targets or dual-targeting strategies (6, 98). Conversely, preclinical data demonstrated reduced toxicity and improved antitumor efficacy in mice receiving CD19-CAR-T treatment following tafasitamab pretreatment, potentially by modulating CD19 antigen density available for CAR-T binding, thereby ameliorating CAR-T cell activation and pyroptosis of lymphoma cells (99).

It remains to be seen when in the treatment sequence allogeneic CAR-T cells may have their highest therapeutic impact. A recent decision by a clinical trial sponsor to deprioritize and terminate their ongoing trial investigating cemacabtagene ansegedleucel (Cema-cel) in a third-line indication reflects this uncertainty. Instead, the sponsor refocused their trial initiatives to pursue a frontline consolidation indication for patients with LBCL who remain MRD positive after completion of a full course of standard 1L induction therapy. This pivotal ALPHA3 trial (NCT06500273) builds on the growing appreciation that both safety and efficacy are improved in patients with low disease burden at the time of infusion.

Conversely, PBCAR19B is being developed as a potential first-in-class therapy specifically for relapse following autologous CD19-CAR-T therapy. This underscores the expanding potential of CAR-engineered products to target unique indications and refine therapeutic sequencing strategies.

Novel CAR-T cell constructs targeting alternative pathways or antigens are also advancing the therapeutic landscape. Transposon-engineered BAFF ligand–based autologous CAR T cells (LMY-920) have demonstrated encouraging responses with a tolerable safety profile in a phase 1 trial (NCT05312801) among 3 patients with r/r B-NHL (100). Notably, the therapy induced a complete response in 1 patient with diffuse large B-cell lymphoma (DLBCL) without causing higher-grade CRS or ICANS. An allogeneic version of the BAFF CAR-T cell is currently under development, which could further expand therapeutic options and improve accessibility for this patient population.

These developments highlight the importance of optimizing the timing and sequencing of CAR-based therapies within the treatment paradigm for LBCL. Biomarker-driven patient selection and strategic placement of CAR-T and CAR-NK therapies in earlier lines of treatment hold significant potential to enhance outcomes and maximize their therapeutic impact.

### Funding of highly innovative cell therapy development programs

Besides ingenuity in cellular engineering, the future of cell therapy programs critically depends on efficient mechanisms of resource allocation to balance risk and reward in a field which has garnered tremendous momentum while it seeks to exploit the promises of precision gene editing.

Of note, the vast majority of included studies in the meta-analysis originated from industry-led cell therapy programs, while academic sponsorship remained limited. While industry funding accelerates commercialization, it also introduces potential bias, which may impinge on scientific independence. US biotech firms in particular benefit from a competitive and mature venture capital ecosystem, while China’s state-backed investments emphasize commercialization. However, venture capital dependence can shift priorities toward short-term gains over high-risk academic innovation.

To balance this, public funding models like the NIH SBIR (Small Business Innovation Research)/STTR (Small Business Technology Transfer) and CPRIT (Cancer Prevention and Research Institute of Texas) emulate venture capital funding mechanics by making high-risk investments and reinvesting financial returns based on revenue-sharing agreements. In contrast, European programs such as IMI (Innovative Medicines Initiative) and EDCTP (European and Developing Countries Clinical Trials Partnership) focus on public-private partnerships but lack the same reinvestment mechanisms that help derisk early-stage clinical assets to enable commercial scale-up. These differences in funding may explain the strong US footprint and present a potential path towards increasing Europe’s presence in the cell therapy space.

### Conclusion

The very low incidences of higher-grade CRS, ICANS, and GvHD across the included studies highlights the exceptional safety profile of allogeneic CAR-T and CAR-NK cell therapies, setting them apart as a safer alternative to autologous CAR-T products. Early efficacy signals are very encouraging, especially considering that this is the first generation of allogeneic cell products. Multi-engineered cell products with additional genetic edits for enhanced functionality are constantly being added to the pipelines of cell therapy development programs and are already being evaluated in the clinic (101). Recent studies leveraging large-scale screening approaches have uncovered previously unrecognized regulators of immune cell function, thereby providing a catalogue of actionable targets to further increase the anti-tumor potency of next-generation cellular therapeutics (86–90, 102).

It will be interesting to see the therapeutic efficacy of allogeneic CAR-T and CAR-NK cells across a wider spectrum of malignancies, including efficacy against solid tumors, where early studies are already underway. Future efforts will need to address how biomarker-driven strategies may refine patient selection and optimize therapeutic outcomes. Ultimately, it will be compelling to observe how various cell therapy programs address the unmet need in r/r LBCL by targeting distinct indications across different lines of therapy and tailoring treatments to patients with unique disease characteristics.

## Data Availability

The original contributions presented in the study are included in the article/**Supplementary Material**. Further inquiries can be directed to the corresponding author.

## Supplementary material

Supplementary Figure 1 Geographical footprint and sponsor type for investigated CAR-IEC products (n=19 unique trials).
